# Inducing Effect of *Corylus avellana* on Cytotoxic Activity in Lung and Breast Cancer Cells via Apoptosis

**DOI:** 10.1007/s11130-024-01198-3

**Published:** 2024-07-01

**Authors:** Ayşegül Çebi, Yalçın Tepe, İmren Alioglu, Ferda Ari

**Affiliations:** 1 Faculty of Health Sciences, Giresun University, Giresun, Turkey; 2 Faculty of Science and Art, Department of Biology, Giresun University, Giresun, Turkey; 3 Faculty of Science and Art, Department of Biology, Bursa Uludag University, Bursa, Turkey; 4 Molecular Biology and Genetic, Democritus University of Thrace, Alexandroupoli, Greece

**Keywords:** *Corylus avellana*, Hazelnut, Breast cancer, Lung cancer, Apoptosis

## Abstract

Turkish hazelnut (*Corylus avellana* L. cv Tombul) is a widely used nut in the chocolate industry and is also rich in polyphenol content, which promises anticancer effects. The anti-cancer and apoptotic effects of hazelnut leaves extracts examined on lung and breast cancer cells. Sulforhodamine B (SRB) and Adenosine 5’- triphosphate (ATP) assays were carried out for cell viability measurement. The mode of cell death was shown morphologically by the double fluorescence staining. Apoptosis was determined by performing caspase-mediated cytokeratin 18 (M30 ELISA) and western blot analysis. PARP, caspase 3, caspase 8, DR4, and GAPHD (Glyceraldehyde-3-phosphate Dehydrogenase) protein bands were visualized as markers of apoptosis. A wound healing test was employed to measure cell migration. Methanol extract of hazelnut leaf exhibited inhibition of cell growth activities in a dose-dependent manner. IC_50_ values were determined as 32.17 µg/ml in MCF-7, 32.16 µg/ml in MDA-MB-231, 20.40 µg/ml in A549 and 12.04 µg/ml in H1299 cells for ethanol extract while it was determined as 21.08 µg/ml in MCF-7, 40.16 µg/ml in MDA-MB-231, 22.04 µg/ml in A549 and 5.91 µg/ml in H1299 cells in methanol extract. In comparison, methanol leaf extracts were more effective in H1299 cells (IC_50_ value was 5.91 µg/ml).In comparison, ethanol leaf extracts were more effective in H1299 cells (IC50 value was 9.722 µg/ml). Western blot analysis demonstrated that hazelnut leaf extract treatment of cancer cells led to cell death via apoptosis and inhibited cell migration in lung and breast cancer cell lines. The cytotoxic effects of hazelnut extract on breast and lung cancer cells might be valuable and promising in elucidating cell death mechanisms for the development of new methods in cancer treatment.

**Supplementary Information:**

The online version contains supplementary material available at 10.1007/s11130-024-01198-3.

## Introduction

Lung cancer, as in the world, is the leading cancer type in our country, diagnosed with approximately 30.000 new cases yearly. Following lung cancer, breast cancer is the second most frequent cancer type all around the globe. Breast cancer is the most common cause of cancer death in women, making up 23% of all female cancers worldwide, with an incidence of 43 *per* 100.000 in Turkey [1, 2].

Despite many new drugs being introduced in cancer treatment each year, the complete treatment of the disease has not been achieved. Multiple extensive searches were carried out for new therapeutics, and the resistance of cancer cells against chemotherapy or other therapies is the most challenging aspect to overcome. For this reason, there is still a dire need for studies towards exploring new compounds to develop novel therapeutics [3].

There are several studies on the growth inhibitory effect of *C. avellana* compound on cancer cells [4–6]. Investigation of cell death in cancerous cells enables understanding of the prognosis and treatment of the disease [7]. Some events that may cause cellular damage, such as oxidative stress caused by the agents used in treatment by triggering apoptosis, can potentially provide treatment of the disease [8, 9]. Unfortunately, most treatments target healthy and cancer cells, resulting in undesirable effects.

Paclitaxel is currently the popular chemotherapeutic medication used in the treatment of lung, breast cancer, and ovarian cancer. Paclitaxel from extracts of defatted and dried hazelnut (*C. avellana L.*) from about 9–70 µg per gram samples was discovered by Hoffman and Shahidi (2009) [10]. The reported paclitaxel amount is about one-tenth of the Pacific yew (*Taxus brevifolia* Nutt.). Nevertheless, hazelnut may still be a new alternative source of taxanes since hazelnut trees grow much faster than the Pacific yew. *C. avellana* extracts were reported to have high activity free radical scavenging properties [11, 12] and the cell suspension culture of *C. avellana* may be a promising alternative for paclitaxel production as Farhadi et al. (2020) recently reported the enhanced paclitaxel biosynthesis and secretion in *C. avellana* cell suspension culture by optimizing elicitors such as fungal cell walls and methyl-β–cyclodextrin. Studies were conducted to increase Taxol production in *C. avellana* culture cells [13, 14]. The main objective of the present study was to assess whether the leaves of *C. avellana* have antiproliferative effects on breast and lung cancer cell lines. The second objective is to determine the molecular mechanism of the antiproliferative effect.

## Materials and Methods

Material and methods are available in Supplementary Material.

## Results and Discussion

### GC–MS Analysis

The GC–MS analysis of *C. avellana* leaf extracts revealed a comprehensive overview of the compounds found in methanol extract. The chromatogram for extract is depicted in Fig. S12. The associated compounds are listed in Table S1, together with their molecular formulas and weights. Extract chromatogram revealed 44 compounds covering 95% of the total area. The GC–MS chromatogram demonstrated that phytol (9.57%), squalene (7.60%), and orcinol (7.32%) are the major metabolic compounds present in the extract of the leaf of *C. avellana*.

### SRB and ATP Viability Assay Results

The inhibitory effects of hazelnut leaf extracts were investigated on human breast cancer (MCF-7, MDA-MB-231) and lung cancer (A549, H1299) cell lines by SRB and ATP assays. According to SRB results; *C. avellana* extracts demonstrated increasing inhibitory effects on human lung cancer lines (A549, H1299) based on dose and time-dependent manner. Ethanol extracts of *C. avellana* of 200 µg/ml were found to be the most effective in both cell lines at the end of 72 h (Fig. S1). Viability percent (%) of breast cancer lines (MCF-7, MDA-MB-231) were decreased with increased dosages of 100 and 200 µg/ml in both ethanol and methanol extracts of *C. avellana.* No significant differences were observed among extracts (Fig. S2).

**Fig. 1:**
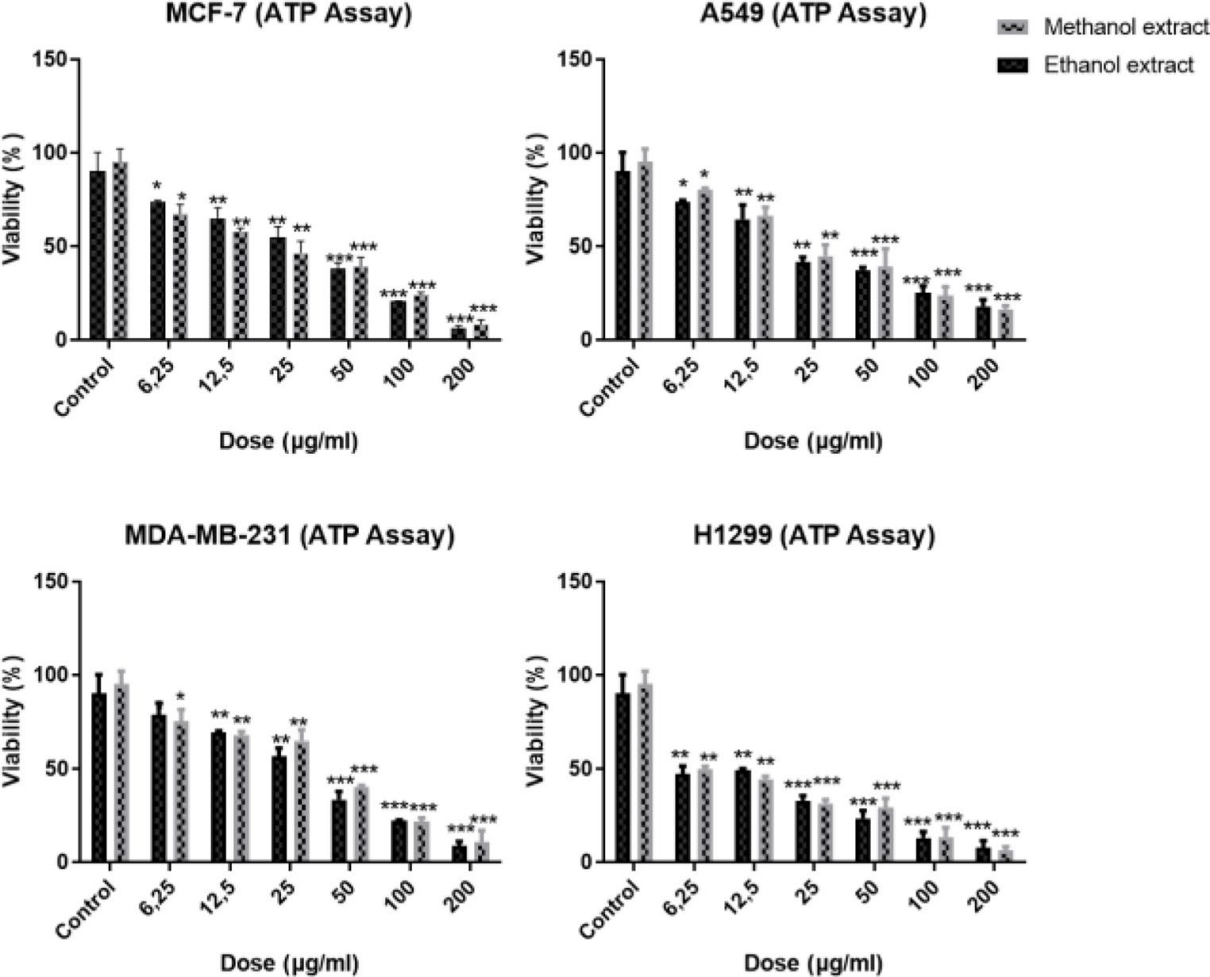
ATP assay results of ethanol and methanol extracts of *C. avellana* on breast and lung cancer cells for 48 h. It was observed that the plant extracts were highly effective and the viability of the cancer cells decreased due to the dose-time. *Denotes statistically significant differences compared to untreated control: *(*p* < 0.05), **(*p* < 0.01), ***(*p* < 0.001). Data are presented as mean ± SD (*n* = 3)

ATP results have shown that starting from the lowest dosage of 6.25 µg/ml, gradually % viability decreases were found in both cancer cells for 72 h, and the most effective dosages were found to be 200 µg/ml for *C. avellana* extracts (Fig. 1).

Significant decreases were observed in the cells’ viability while the concentration of extracts increased. A substantial decline at the dose of 200 µg/ml, observed in all cells, especially in lung cancer cell lines (H1299) at 72 h (*p* < 0.05), demonstrated the most effective inhibition on the cell proliferation.

No antiproliferative effects were observed in hazelnut husk extracts, which may be the result of the high oil content of the husk, which made the extraction process difficult. Therefore, only the leaves of the hazelnut plant were used in the extraction.

Methanol extracts were found to be more effective in breast cancer cells with the IC_50_ values of 21.08 µg/ml and 40.16 µg/ml for MCF-7 and MDA-MB-231 cells, respectively (Table S2). In lung cancer cells, methanol extracts were more effective in A549 with the IC_50_ values of 22.04 µg/ml. On the other hand, ethanol extracts were more effective in H1299 cells with the IC_50_ values of 5.91 µg/ml.

### Cytotoxic Test in Healthy Cells

The cytotoxic effects of different concentrations of ethanol and methanol extracts of hazelnut leaf (6,25–200 µg/ml) on healthy cell lines (Beas-2B and MCF-10 A) were measured by SRB assay for 72 h. Unlike cancer cell lines, *C. avellana* extracts did not show a lethal effect on the healthy cell, and statistically significant reductions were observed with the increasing concentrations (Fig. S3). Selectivity Index (SI) values were determined by the ratio of IC_50_ of healthy cells to cancer cells (Table S3). It was determined that the SI of methanol extracts of *C. avellana* for H1299 lung cancer cells was higher than other cancer cells.

### Fluorescent Dual Staining Results

The dual staining was applied to monitor apoptosis or necrosis states of the cancer cells following *C. avellana* extracts treatment. While Hoechst 33,342 (blue colour) functional to nuclear staining of both living and dead cells with the ability to bind to DNA, PI (red colour) was only able to pass through the damaged membrane and, thus, effective on death or late apoptotic, primer and seconder necrotic cells.

In general, it was shown by Hoechst 33,342 staining that in all cell lines, after the treatment of *C. avellana* extracts, the cell density decreased depending on the dose compared to the control cell lines (Figs. S4-7). In MCF-7 cells, the presence of pyknotic nuclei and Hoechst 33,342 positive/PI negative images after the treatment of methanol and ethanol extracts at doses of 50 µg/ml indicate that the cells are apoptotic, while the presence of Hoechst 33,342 positive/PI positive revealed that it died due to late apoptosis/secondary necrosis (Fig. S4). After 50 and 100 µg/ml treatment of *C. avellana* methanol and ethanol extracts, the presence of pyknotic nuclei in MDA-MB-231 cells compared to the control is determined by Hoechst 33,342 staining, and PI negativity in the same areas indicates that the cells are undergoing apoptosis. At high dose (200 µg/ml) PI positivity cells appear to shift towards necrosis (Fig. S5). Hoechst 33,342 positive/PI positive images of lung cancer cells administered *C. avellana* extracts showed that the cells died dose-dependent manner through late apoptosis/secondary necrosis (Figs. S6-7).

### Cleaved Cytokeratin 18 (M30-Antigen) Results

**Fig. 2:**
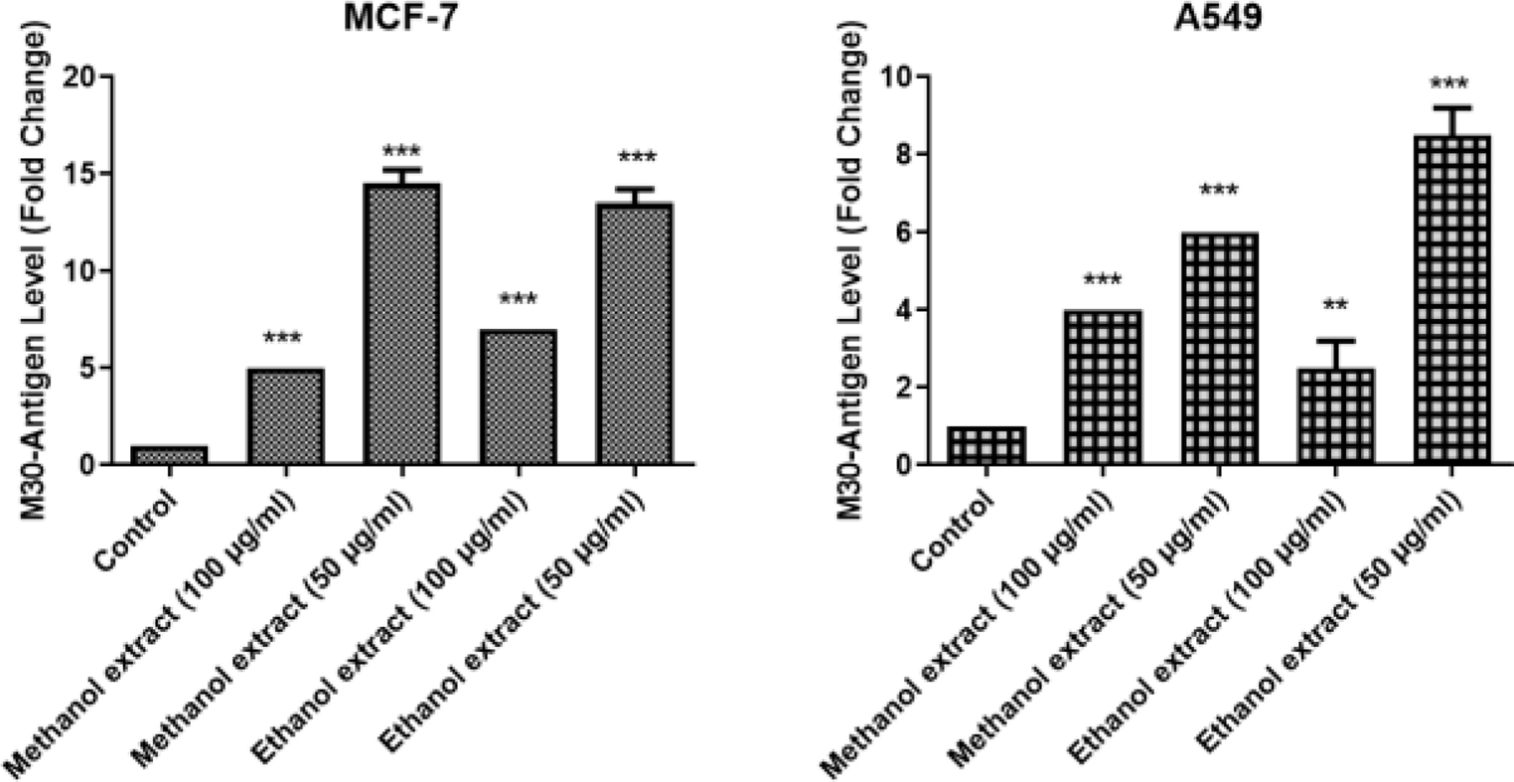
Measurement of caspase-cleaved cytokeratin 18 (M30) levels 48 h after the treatment methanol and ethanol extracts of *C. avellana* in MCF-7 and A549 cells. Paclitaxel was used for control. Statistically meaningful when different doses are compared according to the control (**p < 0.01, ***p < 0.001). Data are presented as mean ± SD (*n* = 3)

As a specific marker for apoptosis, the caspase-cleaved CK18 (M30) scales were done in the concentrations of 50 and 100 µg/ml in order to investigate the cell death mode of MCF-7 and A549 cell lines. The measured absorbance values were calculated by using the standard curve graph and the M30-antigen amounts (U/L). As shown in the Fig. 2, the application of 100 µg/ml and 50 µg/ml concentrations of *C. avellana* extracts caused a statistically significant increase in cancer cells (*p* < 0.05), whereas a less substantial increase in the dose of 100 µg/ml.

As shown in the Fig. 2, the application of 100 µg/ml and 50 µg/ml extracts resulted in a statistically significant increase in cells with a dose of only 50 µg/ml (*p* < 0.05), whereas a less substantial increase at the dose of 100 µg/ml.

M30 results were increased in both cancer cell lines treated with *C. avellana* extracts at 100 and 50 µg/ml doses. Unexpectedly, 100 µg/ml ethanol extracts showed significantly lower M30 values than those of both 100 µg/ml ethanol and methanol extracts (50 and 100 µg/ml) in A549 cells. On the other hand, M30 values of all treatment groups in MCF-7 cells were similar to each other statistically and were found to augment CK18 levels. Except for ethanol 100 µg/ml extract in A549 cells, all M30 values of the present research were close to the positive control, which was evidence of apoptosis.

### Western Blot Results

**Fig. 3:**
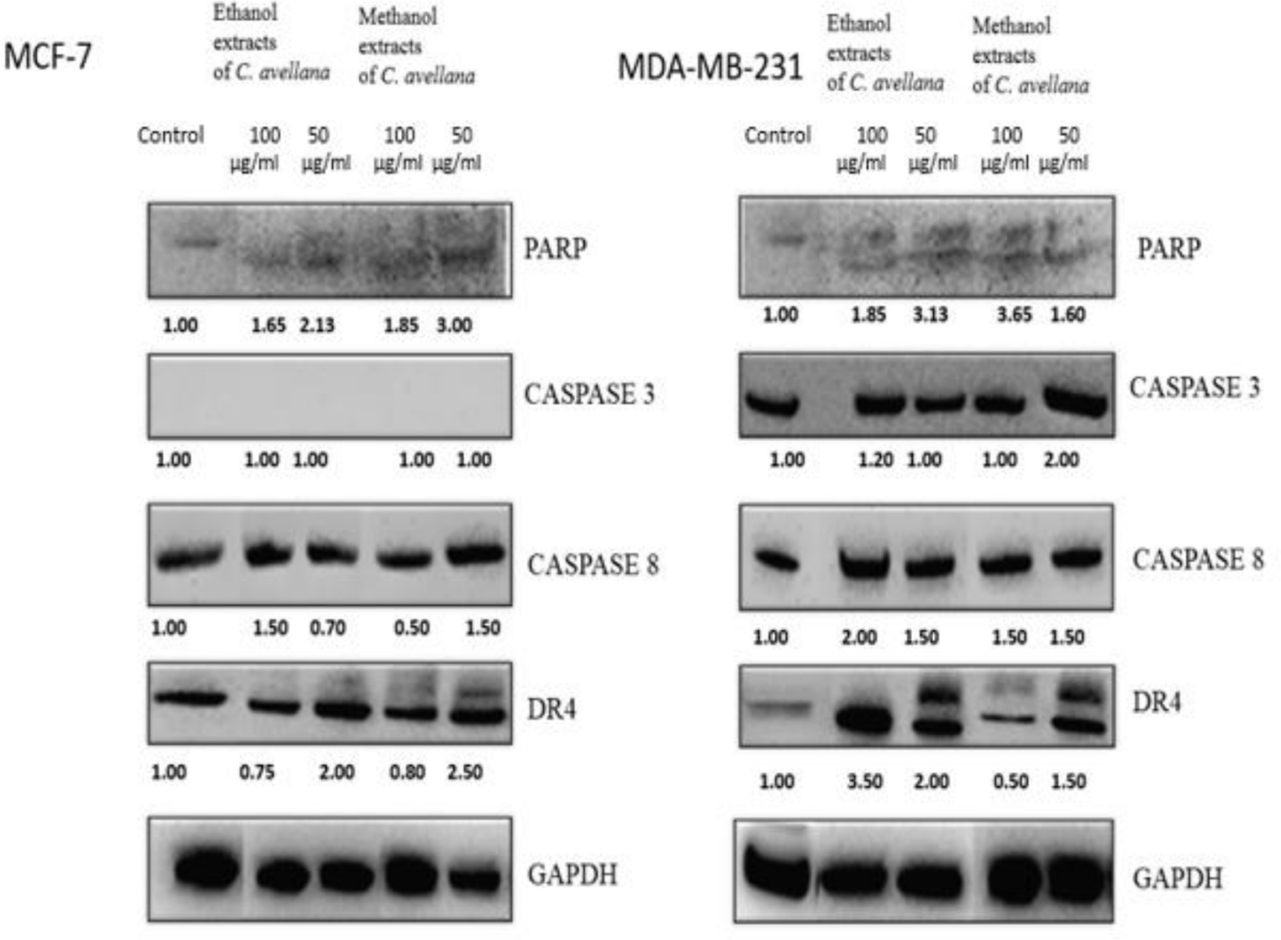
Western blotting analysis of some of the apoptotic proteins in MCF-7 and MDA-MB-231 cells after treatment with methanol and ethanol extracts of *C. avellana* for 72 h. Equal protein loading was confirmed by GAPDH. Densitometry was performed with the ImageJ software and densitometric analysis of the observed bands’ intensity normalized to GAPDH and quantified with respect to controls set to 1.0. Data are presented as mean ± SD (*n* = 3). GAPDH, glyceraldehyde 3-phosphate dehydrogenase; SD, standard deviation

**Fig. 4:**
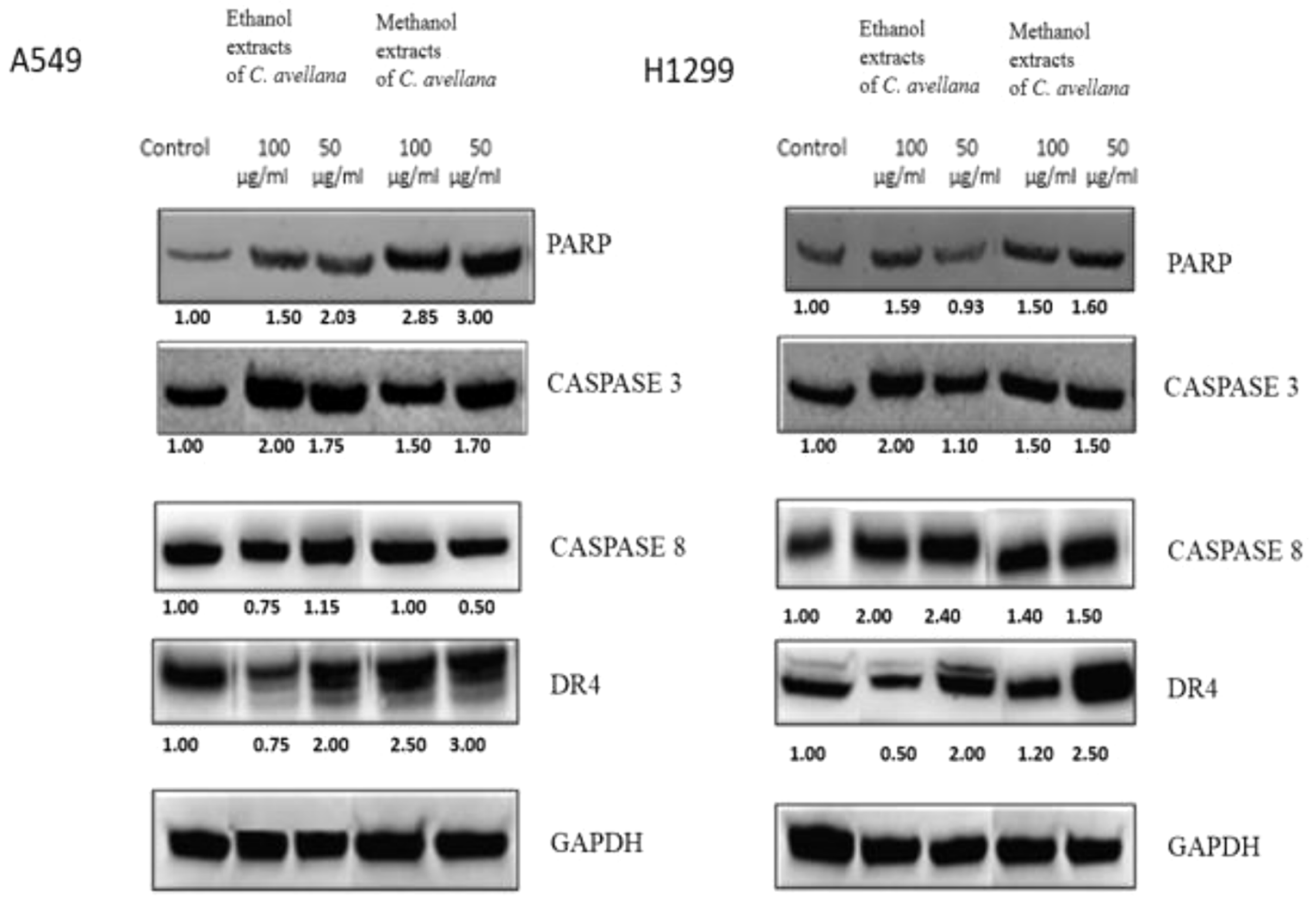
Western blotting analysis of some of the apoptotic proteins in A549 and H1299 cells after treatment with methanol and ethanol extracts of *C. avellana* for 72 h. Equal protein loading was confirmed by GAPDH. Densitometry was performed with the ImageJ software and densitometric analysis of the observed bands’ intensity normalized to GAPDH and quantified with respect to controls set to 1.0. Data are presented as mean ± SD (*n* = 3). GAPDH, glyceraldehyde 3-phosphate dehydrogenase; SD, standard deviation

Western blot was performed to observe the apoptotic proteins such as PARP, caspase 3, caspase 8, DR4, and GAPDH in all cancer cells treated with ethanol and methanol extracts of *C. avellana* at doses of 100 and 50 µg/ml for 72 h (Figs. 3 and 4). GAPDH, a housekeeping protein, was consistently expressed in all treated cells. All apoptotic proteins were detected in the cell lines except caspase 3 in MCF-7 cells which caspase-3 deficient cells.

PARP expression was predominantly observed in A549 and H1299 lung cancer cells treated with both extracts, while it was less obvious in breast cancer cells. Methanol extracts provided clearer PARP images compared to ethanol extracts. *C. avellana* extracts at doses of 100 and 50 µg/ml were revealed to increase PARP expression in all cells significantly compared to the control group. Particularly in MCF-7 and A549 cells, a 3-fold increase compared to the control was noticed at the 50 µg/ml dose of methanol extract (Figs. 3 and 4). Increased caspase 3 expression was observed following extract application in MDA-MB-231, A549 and H1299 cancer cells. The most evident increase (a 2-fold) was evident in MDA-MB-231 cells compared to the control at the 50 µg/ml dose of methanol extract. *C. avellana* extracts of 100 and 50 µg/ml caused an overall 1.5-fold increase in caspase 8 expression of MCF-7 and MDA-MB-231 breast cancer cells. An overall increase of caspase 8 was prominent in A549 and H1299 cells and the most evident increase with almost 2.5-fold was in H1299 cells compared to the control with the dose of 50 µg/ml ethanol extract (Figs. 3 and 4). Increases in DR4 expression were observed in all cells following *C. avellana* extract application. The most significant increase was detected in A549 cells at a dose of 50 µg/ml of methanol extract, a 3-fold increase compared to the control (Figs. 3 and 4). The increase in apoptotic proteins of cancer cells treated with *C. avellana* extracts at doses of 100 and 50 µg/ml is an indication that these extracts cause apoptosis.

### Cell Migration (Wound Healing) Results

The effects of *C. avellana* extracts on the wound healing of MCF-7, MDA-MB-231, A549, and H1299 cells have been performed by cell migration test. All percentages of the wound closure graphs and pictures are shown in Figs. S8-S11. At the end of 12 h, cell migration was not observed treated with ethanol and methanol extracts of *C. avellana* in MCF-7 and MDA-MB-231 cells compared to the control group. On the other hand, in lung cancer cells time depending manner wound healing effects of extracts were varied by cell lines. The methanol extracts of *C. avellana* in A549 cells were not significant in the 6th hour but initiated to exhibit at the 12th hour. In the same cells, while100 µg/ml was ineffective, ethanol extracts were found to be effective only at a 50 µg/ml dose.

Similarly, wound healing effects of ethanol extracts of *C. avellana* on both MDA-MB-231 and MCF-7 cells were statistically evident compared to the control. Wound healing effects of both extracts were observed in all these breast cancer cells starting from the 6th hour. On the other hand, in lung cancer cells time depending manner wound healing effects of extracts were varied by cell lines. The methanol extracts of *C. avellana* in A549 cells were not significant in 6th hour but initiated to exhibit in 12th hour. In the same cells, while 100 µg/ml was ineffective. Ethanol extracts were effective only at a 50 µg/ml dose. The wound healing effects in H1299 cells treated by ethanol extracts of *C. avellana* at both rates were different in the 6th and 12th hours compared to the control. The same cells treated with 50 µg/ml methanol extracts of *C. avellana* were ineffective in the 6th hours and only effective in the 9th hours. The wound healing effect with 100 µg/ml methanol extracts of *C. avellana* was prominent in the 6th and 9th hours.

Moreover, we confirmed the ability of *C. avellana* to inhibit the cell migratory function of breast and lung cancer cells (Figs. S8-S11). The results showed that A549 cells in the control group migrated through the upper well to the lower chamber. The amount of migrated cells was considerably decreased in cells treated with *C. avellana* extracts. The effect of *C. avellana* on cell migration was determined with the aid of a phase contrast microscope.

Hazelnut is a typical snack with high vitamins, minerals, organic acids, lipids (58–64%), protein (11–16%), and carbohydrate (15–18%) content [15] may still be a new alternative source of taxane which cures some cancer types such as lung cancer inhibiting cell proliferation [5]. Some researchers have declared that the cell suspension culture of *C. avellana* may be a promising alternative for paclitaxel production [5, 14, 16]. Methanolic extracts of hazelnut leaves have been confirmed to inhibit the cellular activity of three cancer cell lines of human origin, HeLa, HepG2 (a human hepatocellular liver carcinoma cell line) and MCF-7. The extract of the hazelnut leaves was found to be effective on cervical and liver cancer cells with anti-cancer properties [17]. Hazelnut husk extract was demonstrated to exhibit more toxic and antioxidant effects in T47D-Kbluc (a human breast cancer cell line) and A549 cell lines than normal human gingival fibroblast lines (HGF) [18]. The present study has focused on assessing the antiproliferative effects of the leaves of *C. avellana* and clarifying molecular mechanism of apoptosis on breast and lung cancer cell lines.

The ethanol and methanol extracts are expected to have different effects on cancer cells [19]. In the current study, aforementioned extracts of hazelnut leaves have found to be anti-growth effects in breast cancer cells. MCF-7 cells were found to be more sensitive than the MDA-MB-231 cells with the IC50 values. In lung cancer cells, ethanol extracts were more effective in H1299 cells, while methanol extracts were more effective in A-549 cells with the IC_50_ values. IC_50_ values were determined as 32.17 µg/ml in MCF-7, 32.16 µg/ml in MDA-MB-231, 20.40 µg/ml in A549 and 12.04 µg/ml in H1299 cells for ethanol extract while it was determined as 21.08 µg/ml in MCF-7, 40.16 µg/ml in MDA-MB-231, 22.04 µg/ml in A549 and 5.91 µg/ml in H1299 cells in methanol extract (Table S2). These results show that especially methanol leaf extract is more effective in H1299 lung cells.

Esposito et al. (2017) found that methanol extracts of hazelnut shell had cytotoxic activity in human malignant melanoma cells (A375) and human cervical cancer (HeLa) cells and induced apoptosis in these cells by expression cleaving form of caspase-3 and parp-1 [12]. Both leaves and shell extracts of hazelnut were reported to contain paclitaxel, 10-deacetylbaccatin III, etc. and had some biological activities [4, 20].

According to the double staining results, cancer extract treatment reduced cancer cell density depending on the dose and time. Late apoptosis and secondary necrosis were observed in the cancer cells by staining Hoechst/PI dyes at 200 µg/ml dose. In addition, the presence of pyknotic nuclei and Hoechst 33,342 positive/PI negative images after the treatment of methanol and ethanol extracts at dose of 50 µg/ml indicate that the cells are apoptosis (Figs. S4-7). These results were also supported by the results of M30 antigen, a marker of apoptosis, which significantly increased compared to the control group.

PARP, caspase 3, caspase 8 and DR4 proteins were observed in breast and lung cancer cells treated with ethanol and methanol extracts only with the exception of caspase 3 in MCF-7 cells. Especially at the dose of 50 µg/ml of both extracts show an increase in apoptotic protein expression which revealed to trigger apoptosis in cancer cells.

Methanol extracts provided more dominant PARP images compared to the ethanol extracts. GAPDH proteins were monitored prominently in all the cells treated with extracts (Figs. 3 and 4).

A significant reduction of cell migration was observed in treated cells compared with the control group. Although cell migration at the 24th hour in A549 cells treated with 100 µg/ml ethanol extract was close to the control group, cell migration was significantly lower in cells treated with 50 and 100 µg/ml methanol extract compared to the control group. H1299 cells treated with ethanol extract did not migrate at the 9th hour, whereas the untreated group did. In contrast, the methanol-treated cell group showed cell migration at a rate closer to that of the control. This leads us to conclude that ethanol extract is more effective in H1299 cells (Fig. S9). Although cell migration was low in MDA-MB-231 cells in the group treated with 100 ml of methanol extract, cell migration was higher in the untreated group, and 50 ml of ethanol extract was applied. Therefore, wound healing was higher in control and 50 ml methanol extract groups. This result led us to think that high-dose methanol extract could prevent metastasis in MDA-MB-231 cells. Cell migration at 12th h was not observed in MCF-7 cells treated with ethanol and methanol extracts, while cell migration was observed in untreated cells (Fig. S11). Metastasis is known to be the main problem with cancer and the majority of cancer patient deaths are due to metastasis [21]. In addition to the potential association of mutations with cancer metastasis, it has been reported that both new and more specific therapeutic strategies should be developed to minimize cancer morbidity and mortality in the long term [22]. In this study, it is suggested that the leaf of the hazelnut plant reduces cell migration in lung and breast cancer cells, thus inhibiting metastasis (Figs. S8-11).

New approaches to the treatment of lung and breast cancer are needed. By-products of *C. avellana* could have radical scavenging activity [11] and be an available antioxidant source [23]. Studies have shown that hazelnut can be an alternative anti-cancer plant due to taxol and other bioactive components in its content [5]. In this study, we aimed to elucidate the anti-cancer activity of the leaf of the hazelnut plant by analyzing the molecular mechanism.

## Conclusion

Conventional chemotherapy, surgery and radiation therapy for lung and breast cancer are still inadequate today. Therefore, research on functional foods fortified or enriched with natural potential chemo-preventive food supplements and nutraceuticals that can reduce the incidence of these cancers has gained intense interest recently. The leaf of *C. avellana* plant has been shown to induce apoptosis and reduces migration capacity in lung and breast cancer cells with the current study. Hazelnut, of which Turkey is the world production leader, is an economically valuable chocolate ingredient worldwide. The results of the present study revealed that the leaves of the plant, which indeed are waste product, may be a biotechnologically promising anti-cancer drug raw material.

## Electronic Supplementary Material

Below is the link to the electronic supplementary material.

Supplementary Material 1

## Data Availability

No datasets were generated or analysed during the current study.

## Abbreviations

ATP: Adenosine 5′-triphosphate
GC-MS: Gas chromatography-mass spectrometry
IC_50_: Half-maximal inhibitory concentration
PBS: Phosphate buffered saline
SD: Standard deviation
ANOVA: Analysis of variance
