# Supplementary material for: Inducing Effect of Corylus avellana on Cytotoxic Activity in Lung and Breast Cancer Cells via Apoptosis
Source: Plant Foods Hum Nutr. 2024 Jul 1;79(3):648–55. doi: 10.1007/s11130-024-01198-3 (PMC11410907; doi:10.1007/s11130-024-01198-3)
Supplement: Supplementary file 1 — Supplementary Material 1 [file 11130_2024_1198_MOESM1_ESM.docx]

**Inducing Effect of *Corylus avellana* on Cytotoxic Activity in Lung and Breast Cancer Cells via Apoptosis**

Ayşegül ÇEBİ^a*^, Yalçın TEPE^b^, İmren ALIOGLU^cd^, Ferda ARI^c^

^a^Giresun University, Faculty of Health Sciences, Giresun, Turkey

^b^Giresun University, Faculty of Science and Art, Department of Biology, Giresun Turkey

^c^Bursa Uludag University, Faculty of Science and Art, Department of Biology, Bursa, Turkey

^d^Democritus University of Thrace, Molecular Biology and Genetic, Alexandroupoli, Greece

* Corresponding author.

Ayşegül ÇEBİ, cebiaysegul@hotmail.com

Phone: +90-5434351255

**Material and Method**

*2.1. Cell Culture, Treatments with Compounds*

*2.1.1. Sample Collection and Extraction*

Hazelnut leaf samples and fruit husks were collected from the Giresun province of Turkey during the harvest season in August 2018. All samples were dried in an oven at 37 °C for 48 hours or until complete dryness. The dried samples were ground with a grinder then, they were passed through a 0.85 mm sieve. The samples were stored in the refrigerator in zipped bags until extraction. Two different solvents were used for extractions, either ethanol or methanol. The powdered samples of 25 g were taken separately to perform an extraction with 250 ml of ethanol or methanol in the soxhlet device for 5 hours. Ethanol and methanol extracts were then evaporated. Lyophilisates were obtained by lyophilization within 24 hours and stored at -20^°^C until further analysis [1].

*2.1.2. GC-MS Analysis*

Analysis of hazelnut leaf content was performed with the aid of a GS-MS (Agilent Model 7890 A/5975 C inert MSD) device with a SIM acquisition mode. The flow rate of the GS-MS, whose carrier gas is helium, is 1.5 mL min^−1^. Only 1 µL of sample was run in pulsed split mode. The silox column (30 m x 250 µm x 0.25 µm) (an Agilent 5 % HP-5MS phenyl methyl) had a flow rate of 1.5 mL min^−1^, a mean velocity of 44.635 cm sec^−1^, with 12.675 psi pressure. The injection port’s temperature was initially 50 °C, then at a 5 °C/min rate, set to 75 °C for 5 min., 150 °C for 5 min., 240 °C for 10 min. and to finally rise to 325 °C. Total run time was 63 min [2].

*2.1.3. Chemicals and Cell Culture*

Human breast cancer cell lines (MCF-7 and MDA-MB-231) were cultured in RPMI 1640 medium containing 1% glutamine at 37 ^°^C, 5% CO_2_ in media containing 1% penicillin-streptomycin (10.000 U/ml penicillin, 10 mg/ml streptomycin) and 5% fetal bovine serum (FBS). Human lung cancer cell lines (A549, H1299) were cultured in RPMI 1640 medium containing 1% glutamine and 1% non-essential amino acids at 37 ^°^C, 5% CO_2_ in media containing 1% penicillin-streptomycin (10.000 U/ml penicillin, 10 mg/ml streptomycin) and 10% FBS. Cells were obtained from the American Type Culture Collection (Rockville, Maryland).

Stock solutions of *C. avellana* lyophilisate were prepared with dimethyl sulfoxide (DMSO) and stored at -20 °C. Lyophilisates, dissolved at different concentrations, were introduced in culture media. DMSO ratio at the highest concentration did not exceed 0.01%.

*2.2. Cell Viability Assays*

*C. avellana* leaf extracts were introduced into cell lines for 48-72 hours in different

treatment rates. Six treatment with two replications each within the doses of 6.25, 12.5, 25, 50,100 and 200 μg/ml rates were performed. Viability assays were carried out in 96-well "flask" cell culture vessels. Each dose was tested using three different wells. Two independent experiments were performed simultaneously.

*2.2.1. Sulforhodamine B (SRB) Assay*

The SRB method is based on the ability of SRB to bind to the protein content of cells fixed with trichloroacetic acid (TCA; AppliChem, Germany) in mild acidic conditions. By dissolving in the basic medium, SRB allows a colourimetric measurement based on the amount of cells. For SRB testing, MCF-7, MDA-MB-231, A549 and H1299 cells were used after cultivation in 96-well cell culture dishes at a density of 2500-7500 cells per 100 µl well. After 24 hours of incubation, lyophilizes were added at various concentrations (6.25-200 µg/ml) for 48 and 72h.

After 48 hours of incubation, the plate was inverted and discarded, and 100 µl of 10% TCA were pipetted into each well. The plate was left for an hour at 4°C, and the contents were discarded and washed five times with 200 µl deionized water. Pipette 100 µl of 0.4% (w/v) SRB solution into each well, 30 minutes incubation at room temperature in the dark, and washed five times with 100 µl of 1% acetic acid solution. After the wells dried completely, Tris base (pH: 10, 100 µl, 10 mM) was pipetted into each well. The plate was placed at 600 rpm for 10 minutes in the shaker. Optical density (absorbance) was measured in an ELISA microplate reader at a wavelength of 564 nm. Cell viability was calculated by the formula below [3].

Cell viability (%) = [100 × (Sample absorbance) / (Control absorbance)]

*2.2.2. Adenosine 5′‐triphosphate (ATP) Assay*

The principle of this method is based on measuring the intracellular ATP content in cells grown in cell culture [4]. As ATP level measurement is based on luminescence technology, it is much more sensitive than other viability assays, and even at low cell numbers (up to 20 cells). The method has a perfect correlation between the number of live cells and the relative light unit values (RLU) read. Therefore, it is more sensitive and reliable than colorimetric tests (such as MTT, MTS and XTT). Therefore, our laboratory always uses these two tests together to check the accuracy of SRB viability test results by ATP test.

The lyophilisates, which were found to be effective as a result of the SRB test, were applied to 96-well cell culture dishes. The cells (MCF-7, MDA-MB-231, A549, H1299) at different concentrations (6.25-200 µg/ml) were counted, and 5x10^3^ cells were seeded to each well in 100 µl medium. Cells were incubated in an oven at 37 ^0^C, 5% CO_2_ for 48 hours. ATP content in the cells was measured using the luminometer (Bio-Tek, USA) with the luciferase-luciferase bioluminescence reaction shown below.

Results were taken as a relative light unit (Relative Light Unit, RLU) and expressed as U/l. Thus, the cytotoxic/cytostatic effects of the compounds were confirmed according to the RLU values to be obtained from the treated and non-treated cells. This method was performed using the "Adenosine 5′-triphosphate (ATP) bioluminescent somatic cell assay kit" (Sigma, MO, USA).

*Lusiferase*

ATP + Luciferin + O_2_ →→→→→→→→ AMP + 2Pi + CO_2_ + Photon (RLU)

Thus, the percentage viability of the samples was calculated according to the RLU values that were treated and obtained from the control cells. The viability calculation was calculated as follows.

Viability (%) = [100 x (Sample RLU) / (Control RLU)]

Subsequently, IC50 (half-maximal inhibitory concentration) values were calculated according to the viability (%) results.

*2.3. Viability Test in Noncancerous Cell lines*

This method investigates the toxic effects of hazelnut ethanol and methanol extracts in healthy cells (BEAS-2B and MCF-10A). SRB assay was also performed in healthy cells to observe the possible cytotoxic effects of the hazelnut extracts.

To fully evaluate the anti-proliferative activity of the extracts, the selectivity index was determined by the ratio of IC50 of healthy cells to cancer cells. Selectivity Index (SI) values were calculated for this purpose using the following formula:

SI: (IC50 of healthy cells) / (IC50 of cancer cells)

*2.4. Fluorescent Staining Method with Hoechst 33342 and Propidium Iodide*

Nucleus morphology and membrane integrity were examined by the double staining method. Two different fluorescent dyes were used to determine the effect of lyophilizes on cell death mode (apoptosis/necrosis): Hoechst 33342 (blue colour) is a dye that can bind to DNA and thus penetrate through the cell membrane. It is used to stain the nuclei of living and dead (apoptotic/necrotic) cells. Propidium iodide (PI) (red colour) is a fluorescent nucleic acid dye that can only penetrate membrane-damaged cells, so that it can stain all the dead cells (late apoptotic/secondary necrotic or primary necrotic). PI dye is not effective on living cells as expelled by them [1]. The cells treated with either ethanol or methanol extracts of hazelnut (6.25-200 µg/ml) for 72 hours were stained with Hoechst 33342-PI solution for 20 min and analyzed by fluorescence microscope for viability.

When marked with a fluorescent agent, the apoptotic cells can be visualized and examined under fluorescence microscopy. The effect of different concentrations of extracts (200, 100 and 50 μg/ml) on the cells at 72 hours was evaluated under a fluorescence microscope using Hoechst 33342 and Propidium Iodide.

*2.5. Caspase-cleaved Cytokeratin 18 (M30-Antigen) Assay*

M30-Antigen levels were examined to determine extracts' cell death mode (apoptosis or necrosis). Cytokeratin 18 (CK18), an essential protein of the cytoskeleton, goes out of the cell during cell death [5]. In cells that undergo apoptosis, CK18 (CK18-Asp396) occurs by cleaving the CK-18 and the effect of caspase, which enzyme group activated only in apoptotic cells. M30 monoclonal antibody provides the use of CKs as an apoptotic marker, especially by recognizing CK18's fragment (M30 antigen) broken in Asp396. Thus, the broken CK18 ELISA method, which is a marker specific to apoptosis, is detected. When the M30 antibody recognizes this special CK18 and is introduced into the apoptotic cells, the presence of apoptosis is measured.

The possible presence of apoptosis in cancer cells which treated the lyophlysates at various concentrations was determined by the M30 Apoptosense ELISA kit (M30-Apoptosense ELISA kit, Peviva, Sweden).

MCF-7 and A549 cells were counted and seeded into 200 μl 96-well cell culture dishes, three replicates each, then incubated for 72 hours with 100 and 50 μg/ml extracts of hazelnut leaf 37 ^°^C, 5% CO_2_. At the end of the treatment period, the cells were lysed with 10% NP-40 (v/v) for 10 min. The content of identical wells was pooled and centrifuged at 2000 rpm for 10s to remove the debris. All samples were placed into wells coated with a mouse monoclonal antibody as a catcher. After washing, horseradish peroxidase-conjugated antibody (M30 antibody) was used for detection. The absorbance was determined with an ELISA reader at 450 nm.

*2.6. Identifying Apoptosis Markers with Western Blot*

Cell lysates were prepared as previously described [3]. MCF-7, MDA-MB-231 breast cancer and A549 and H1299 lung cancer cells were seeded in 75 cm^2^ flasks. When the cells reached 70% confluency was plated in 6 well plates (12,5–200 μg/ml) and treated with extracts of hazelnut leaf for 72 h. Following 72 h incubation, the supernatants of the extracts applied plates were collected in 15 ml of falcon tubes and the supernatant of the negative control flask was removed. Cells were removed by scraper (Corning) and transferred to tubes. The suspension was centrifuged at +4°C for 5 minutes at 1000g. Then, the supernatants were removed, and the lysis buffer was pipetted into pellets. Protein was extracted using the RIPA Lysing Buffer System (Santa Cruz Biotechnology, Dallas) according to the manufacturer's protocol. Falcon tubes were kept on ice for 30 minutes in the dark. At the end of the period, the solutions were transferred to 1.5 ml tubes and centrifuged at +4°C for 10 minutes. Supernatants were collected in 0.5 ml tubes. Protein concentration was determined with the BCA Protein Assay Kit (Thermo Fisher Scientific, CA). Equal concentrations of proteins were subjected to 12% sodium dodecyl sulfate-polyacrylamide gel electrophoresis. It was then transferred to a nitrocellulose membrane. Membranes were incubated with the following primary antibodies overnight at 4°C [6].

The membranes were probed sequentially with PARP, caspase 3, caspase 8, DR4 and GAPDH antibodies (1:1000 dilution; Cell Signaling Technology, MA, USA). Then for the second antibody incubation incubated with HRB-linked anti-rabbit IgG antibodies (1:2000 dilution; Cell Signaling Technologies, MA, USA or 1:5000 dilution; Amersham Biosciences, Sweden). Antibodies bound to membranes were visualized using the Fusion FX-7 imaging device system. The membrane-specific antibody was introduced with a secondary antibody that identifies the antibody. Finally, it was provided that the target protein was displayed on the membrane by means of light generated as a result of *C. avellana* extracts treatment of mentioned enzyme with the substrate.

*2. 7. Cell Migration (Wound Healing) Test*

Monolayer wound healing analysis was performed to determine the cell migration rate. A549, H1299, MDA-MB-231 and MCF-7 cells were cultivated in 6-well cell culture dishes when cells were in 80% confluence washed with 1x PBS.

After a wound line was then created in 6-well cell culture dishes, the culture was created with a pipette tip evenly and flatly on the surface of the containers. Then first added to the control group cells medium and to the other cell, wells added ethanol and methanol extracts in hazelnut as 10 and 50 μg/ml. The cell migration was monitored by a phase-contrast microscope in a time-course manner, and distances were analyzed. The moment of wound creation was considered as the beginning (0^th^) and healing were recorded from the same point at throughout the 24 hr treatment period. The densitometric analysis was carried out with Image-J. After normalization, the migration rate (%) of cells was determined depending on dose and time [7].

*2.8. Statistical Analysis*

Results were presented as ±SD (standard deviation) by calculating the mean of at least three independent studies. All statistical analyzes were tested with one-way analysis of variance (ANOVA) and evaluated with GraphPad 9.12 program. P < 0.05, P < 0.01 and P < 0.001 values were considered statistically significant. Additionally, the microscope images were meticulously analyzed and quantified using the ImageJ software program.


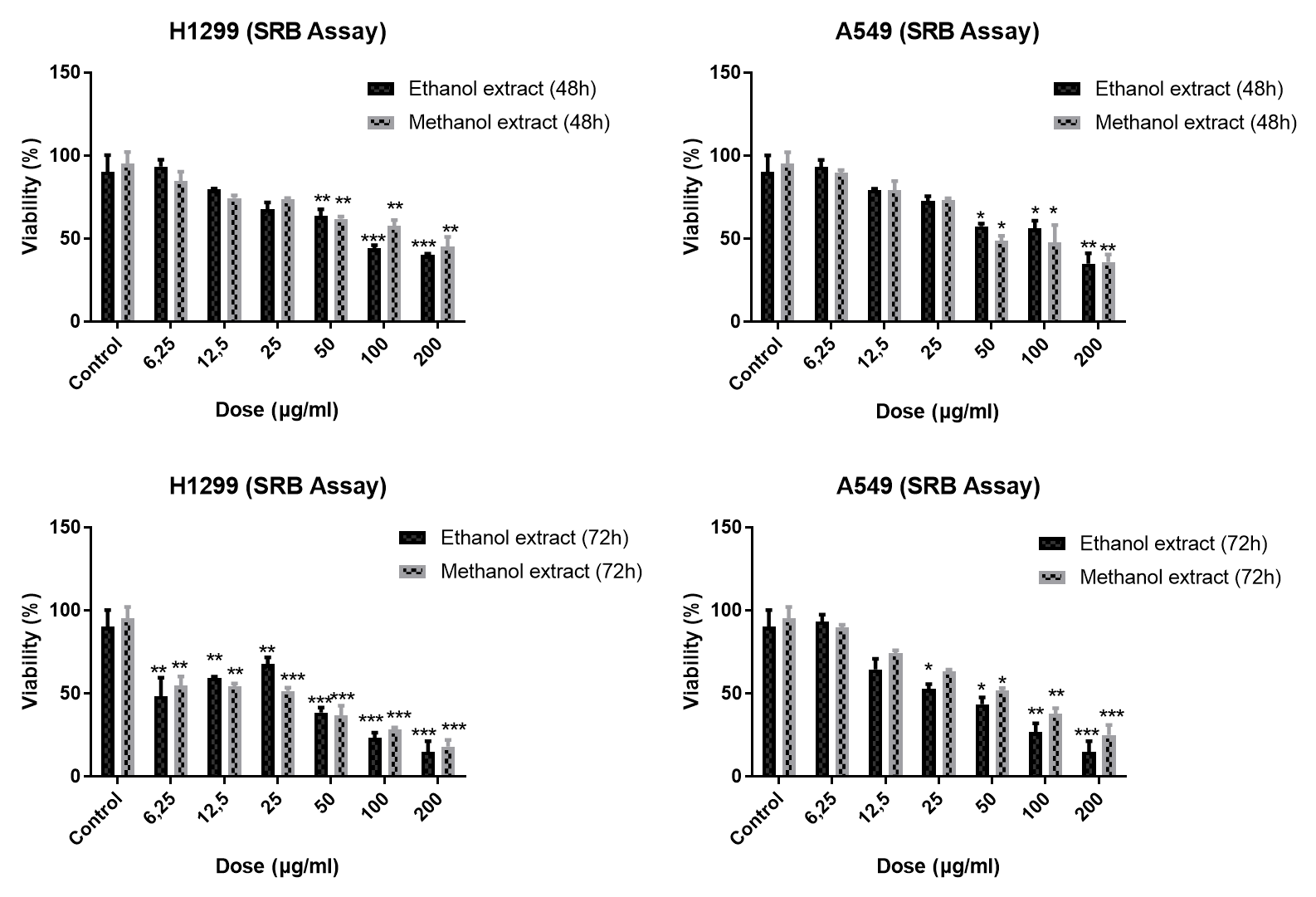
 **Fig. S1** SRB assay results of ethanol and methanol extracts of *C. avellana* on lung cancer cells (H1299 and A549) for 48 and 72h. *Denotes statistically significant differences compared to untreated control: *(p<0.05), **(p<0.01), ***(p<0.001). Data are presented as mean±SD (n=3)


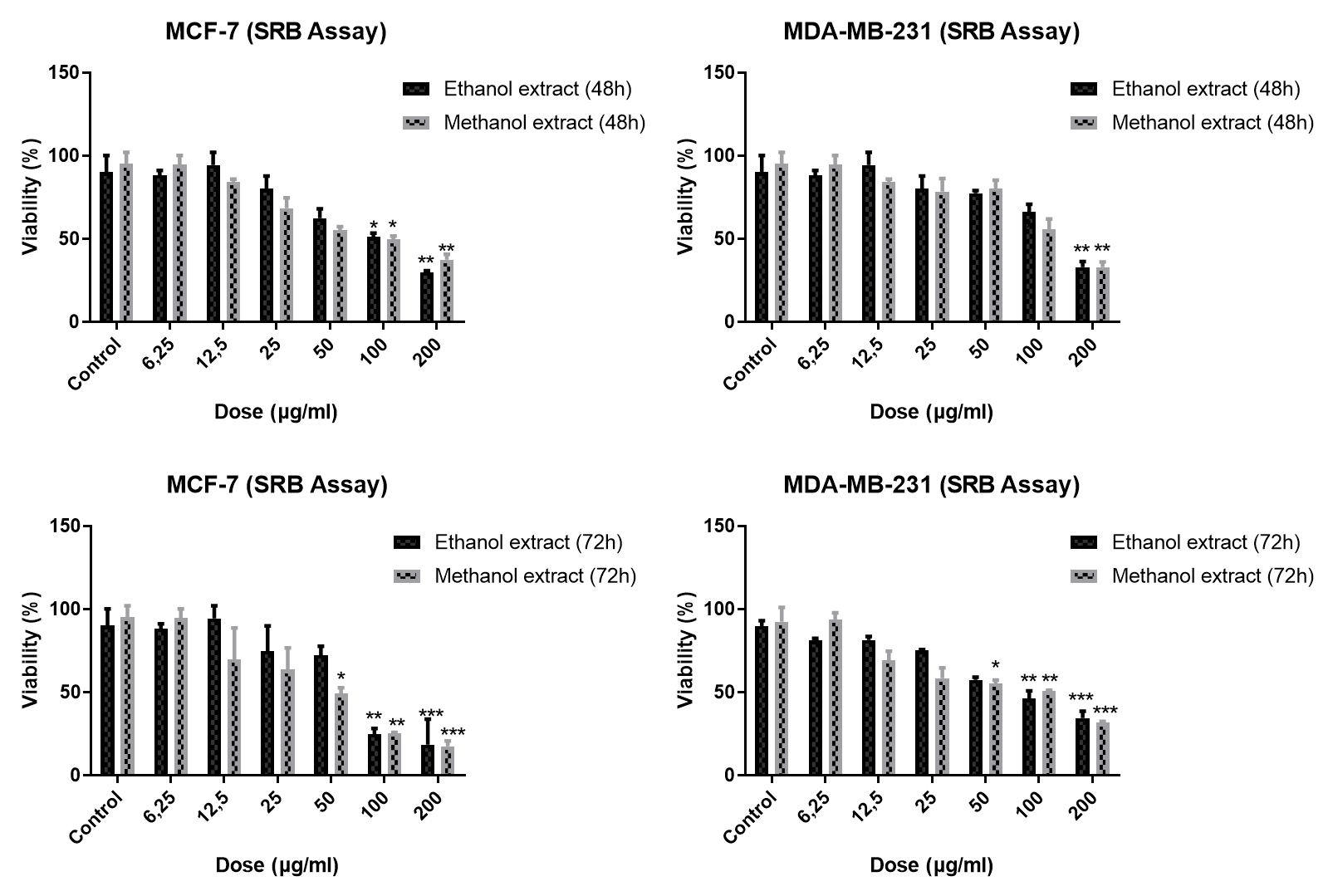


**Fig. S2** SRB assay results of ethanol and methanol extracts of C. avellana on breast cancer cells (MCF-7 and MDA MB 231) for 48 and 72h. *Denotes statistically significant differences compared to untreated control: *(p<0.05), **(p<0.01), ***(p<0.001). Data are presented as mean±SD (n=3)


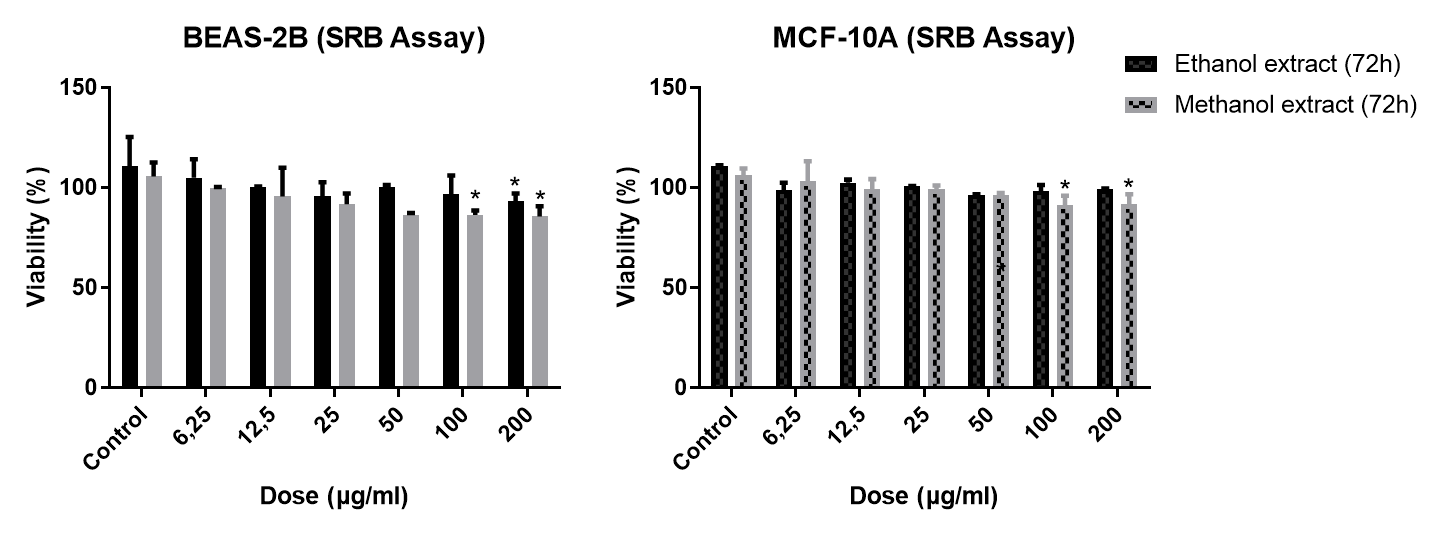


**Fig. S3** SRB assay results of ethanol and methanol extracts of C. avellana on healthy lung (BEAS-2B) and breast (MCF-10A) cells for 72h. *Denotes statistically significant differences compared to untreated control: *(p<0.05). Data are presented as mean±SD (n=3)


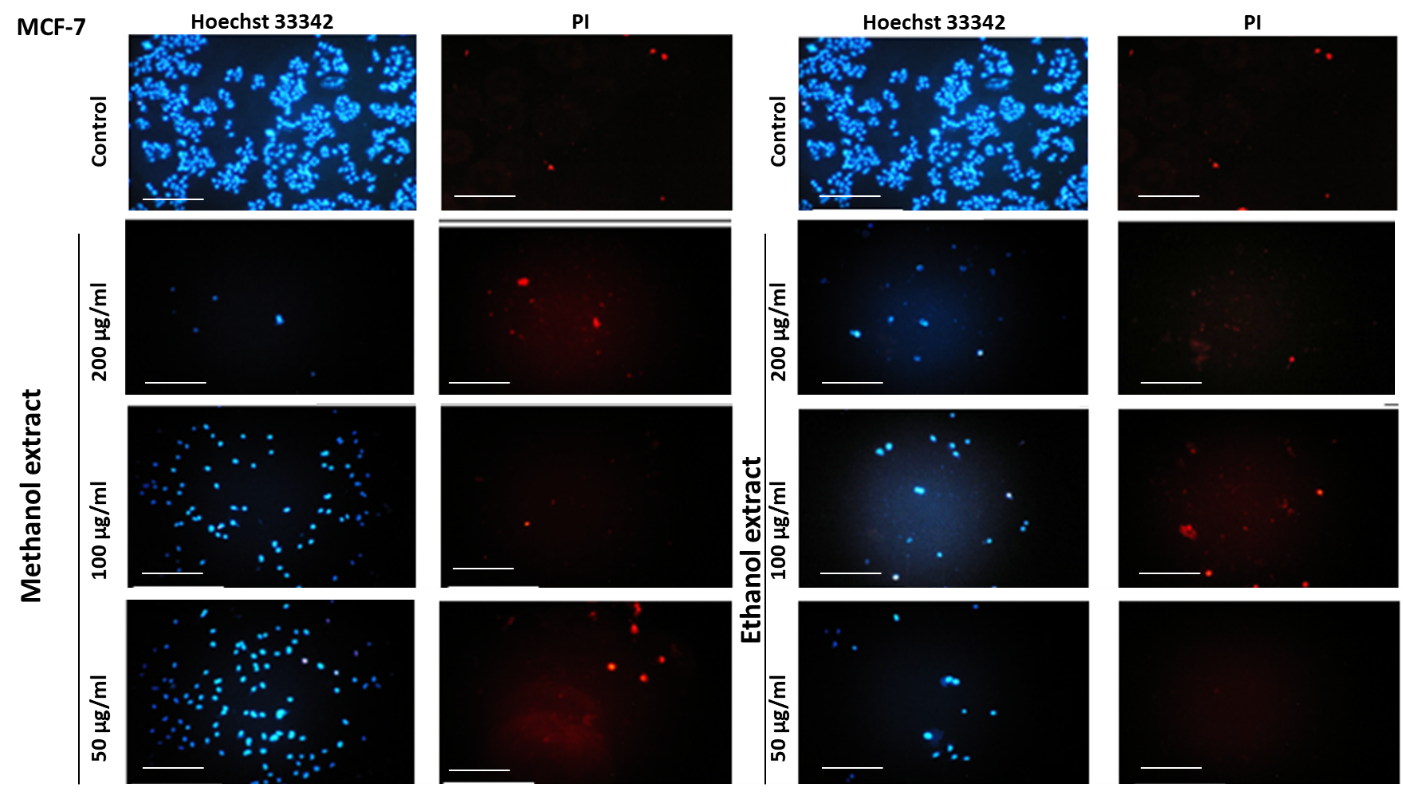
**Fig. S4** Fluorescent images of Hoechst 33342 (blue labeled) and Propidium iodide (PI, red labeled) after treatment with methanol and ethanol extracts *C. avellana* in MCF-7 breast cancer cells for 72 hr. Microscope objective magnification is ×20 and the scale bar length is 50 μm

**
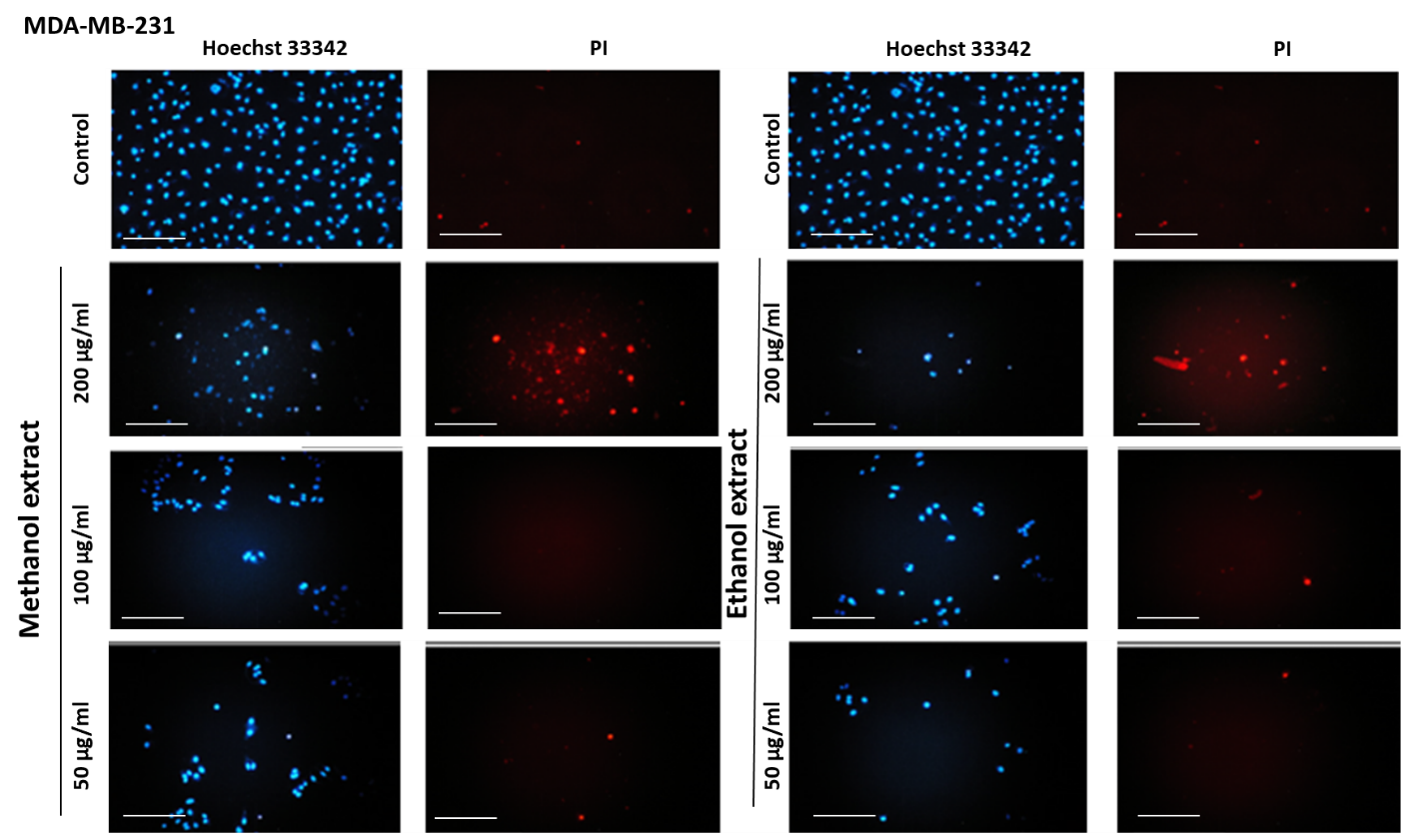
**

**Fig. S5** Fluorescent images of Hoechst 33342 (blue labeled) and Propidium iodide (PI, red labeled) after treatment with methanol and ethanol extracts *C. avellana* in MDA-MB-231 breast cancer cells for 72 hr. Microscope objective magnification is ×20 and the scale bar length is 50 μm


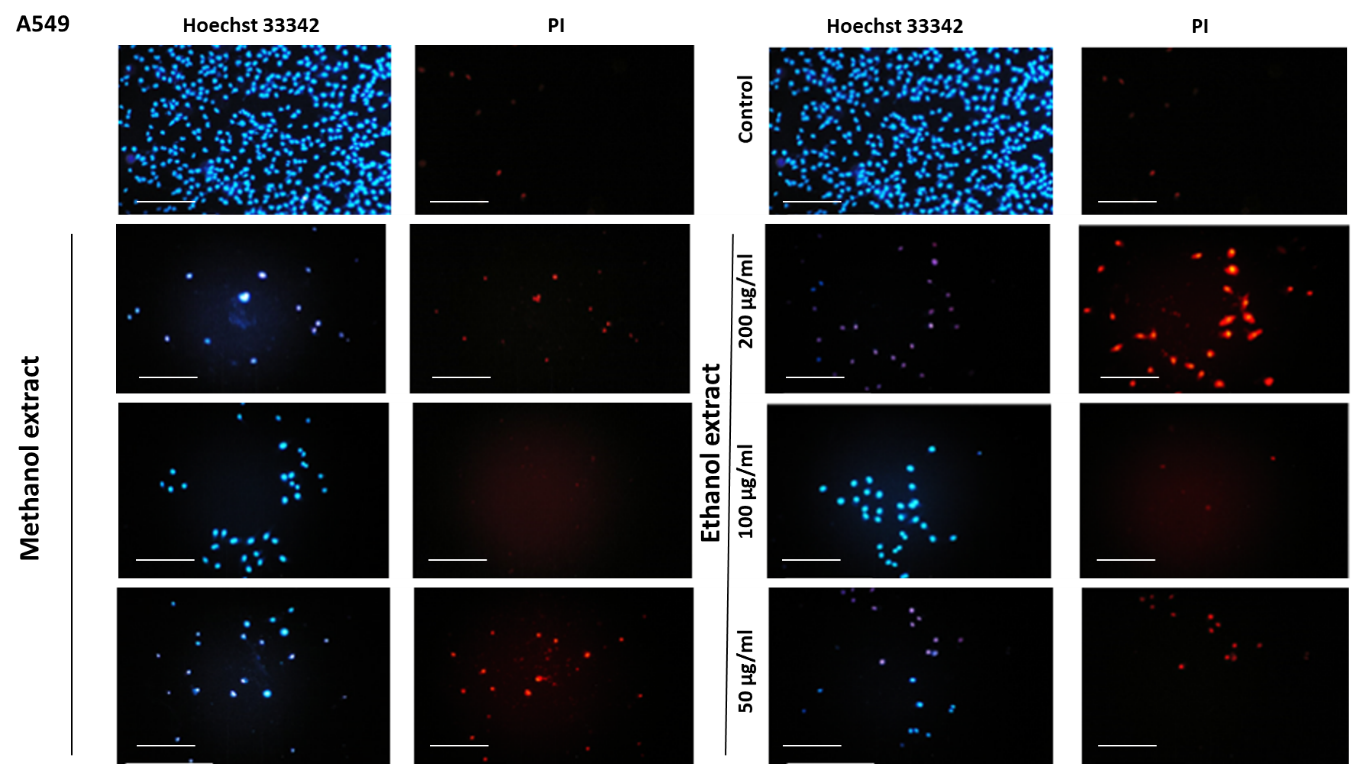


**Fig. S6** Fluorescent images of Hoechst 33342 (blue labeled) and Propidium iodide (PI, red labeled) after treatment with methanol and ethanol extracts *C. avellana* in A549 lung cancer cells for 72 hr. Microscope objective magnification is ×20 and the scale bar length is 50 μm


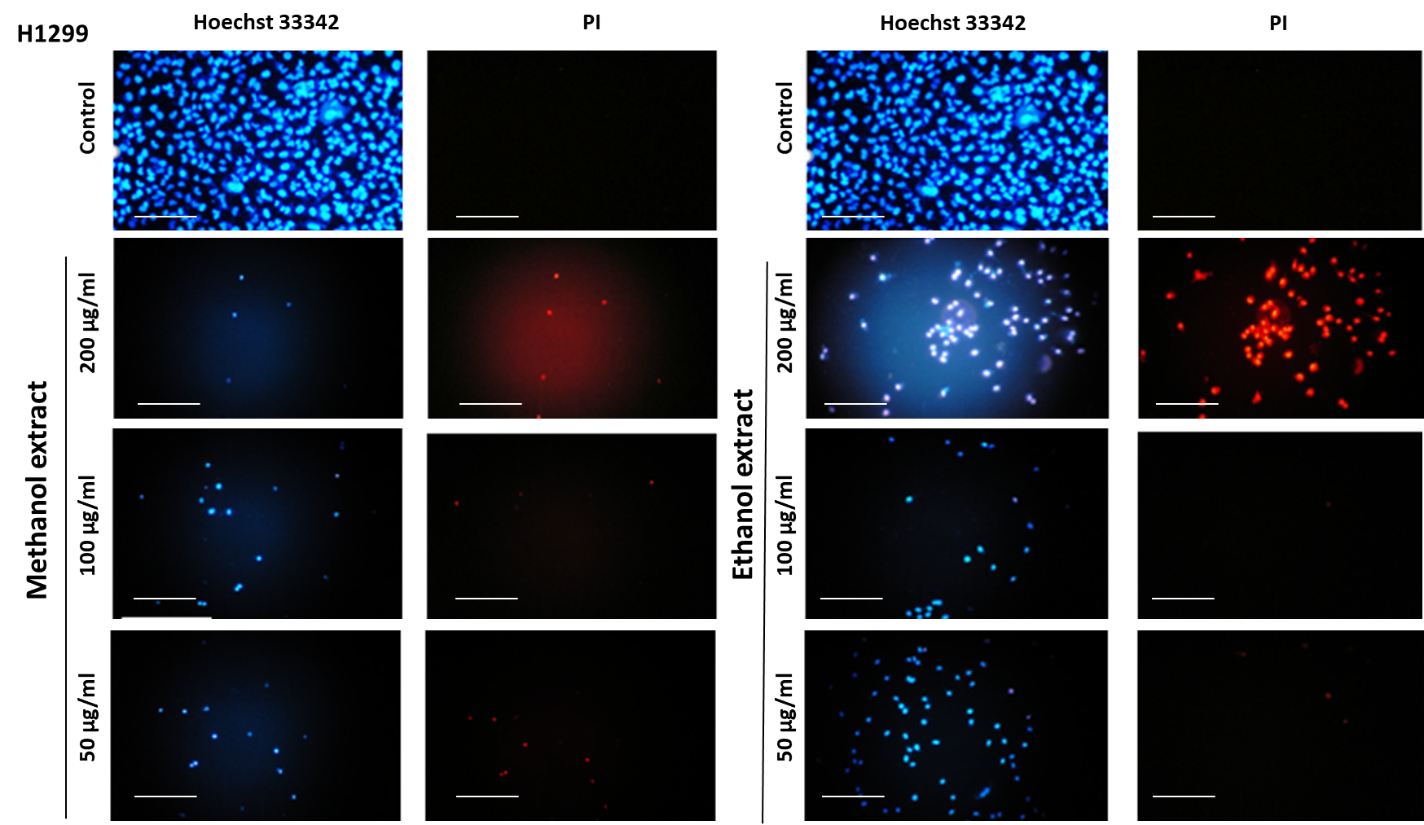


**Fig. S7** Fluorescent images of Hoechst 33342 (blue labeled) and Propidium iodide (PI, red labeled) after treatment with methanol and ethanol extracts *C. avellana* in H1299 lung cancer cells for 72 hr. Microscope objective magnification is ×20 and the scale bar length is 50 μm


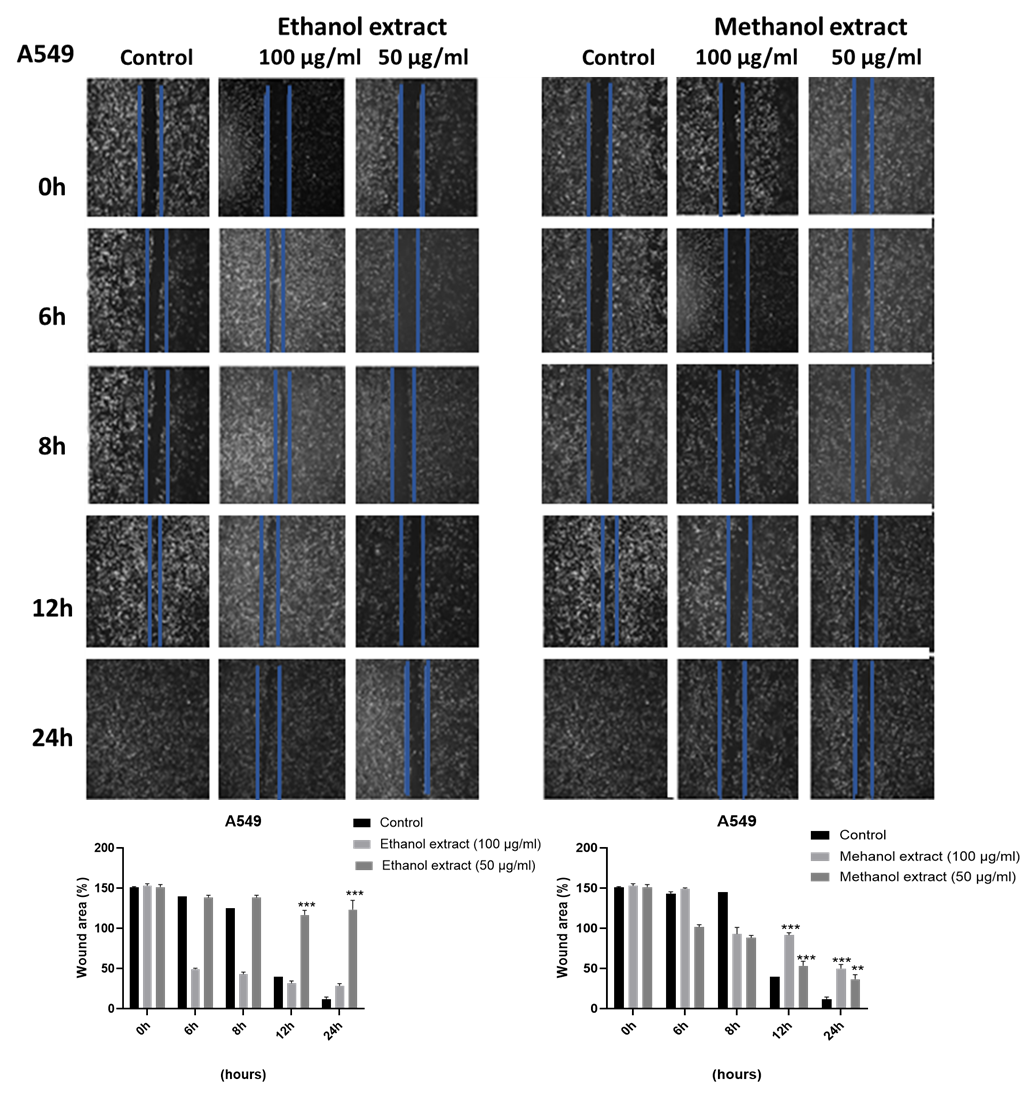


**Fig. S8** Cell migration evaluated by wound-healing assay after treatment with methanol and ethanol extracts of *C. avellana* on A549 cells up to 24 hours. The cell images were taken at 0h, 6h, 8h, 12h, 24h. The wound with of views was measured, and the healing width was calculated by wound with at 0 h time point minus wound with at 24 h time point and normalized by control. *Indicates statistically significant differences compared to the control group cells at the same time period **(p<0.01), ***(p<0.001). Data are shown as mean ± SD (*n*=3). Microscope objective magnification ×4


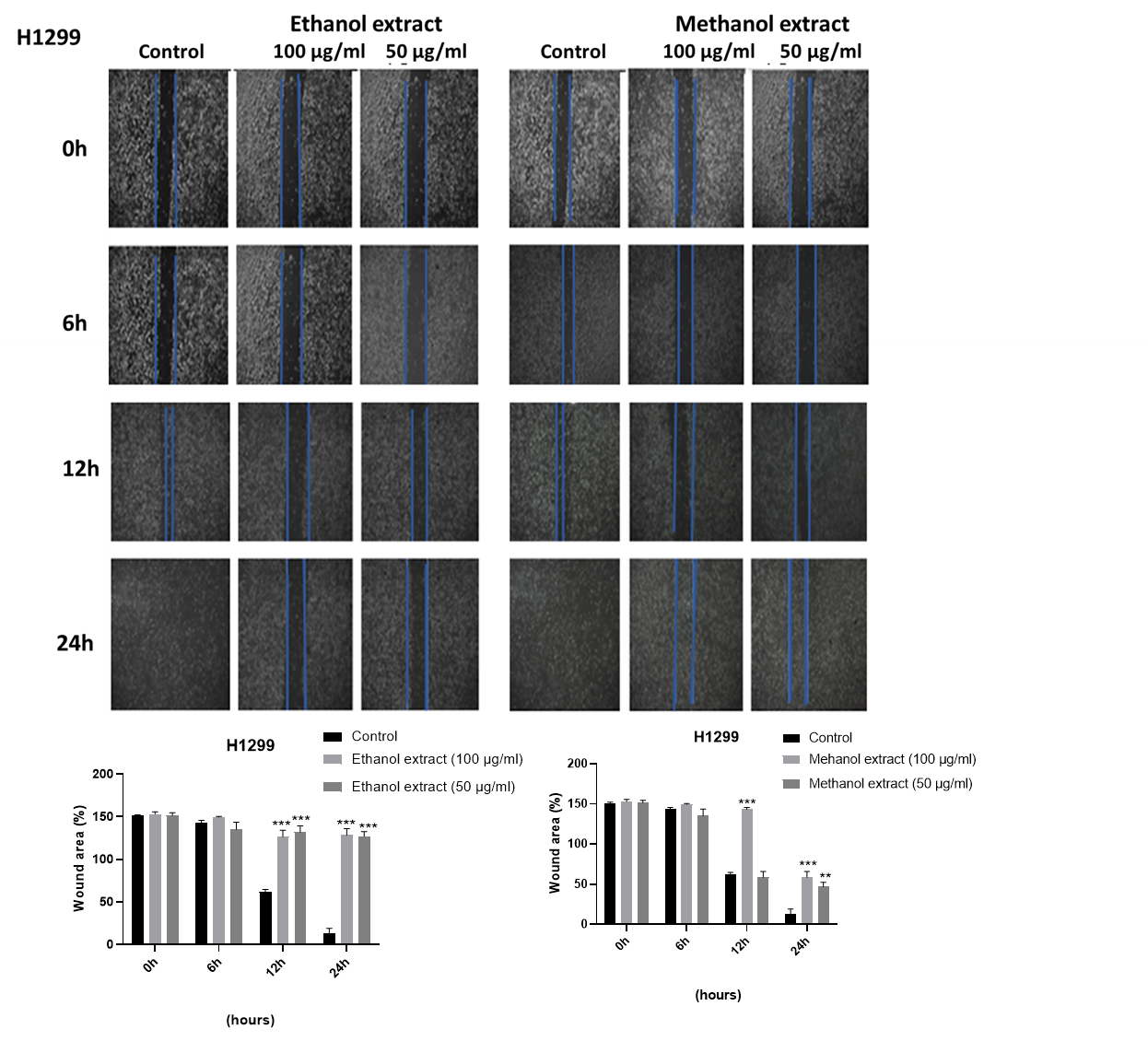


**Fig. S9** Cell migration evaluated by wound-healing assay after treatment with methanol and ethanol extracts of *C. avellana* on H1299 cells up to 24 hours. The cell images were taken at 0h, 6h, 12h, 24h. The wound with of views was measured, and the healing width was calculated by wound with at 0 h time point minus wound with at 24 h time point and normalized by control. *Indicates statistically significant differences compared to the control group cells at the same time period **(p<0.01), ***(p<0.001). Data are shown as mean ± SD (*n*=3). Microscope objective magnification ×4


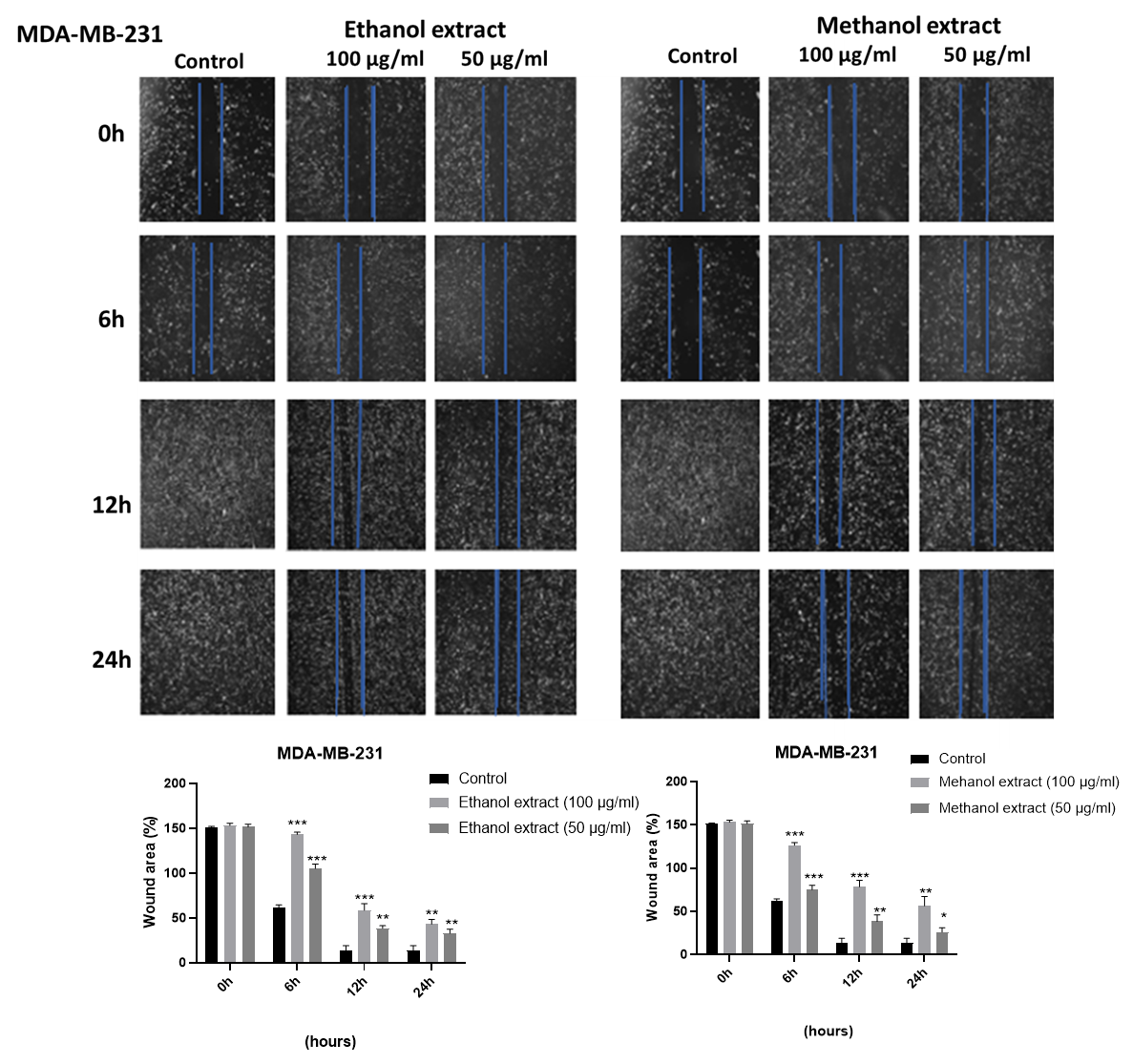


**Fig. S10** Cell migration evaluated by wound-healing assay after treatment with methanol and ethanol extracts of *C. avellana* on MDA-MB-231 cells up to 24 hours. The cell images were taken at 0h, 6h, 12h, 24h. The wound with of views was measured, and the healing width was calculated by wound with at 0 h time point minus wound with at 24 h time point and normalized by control. *Indicates statistically significant differences compared to the control group cells at the same time period *(p<0.05), **(p<0.01), ***(p<0.001). Data are shown as mean ± SD (*n*=3). Microscope objective magnification ×4


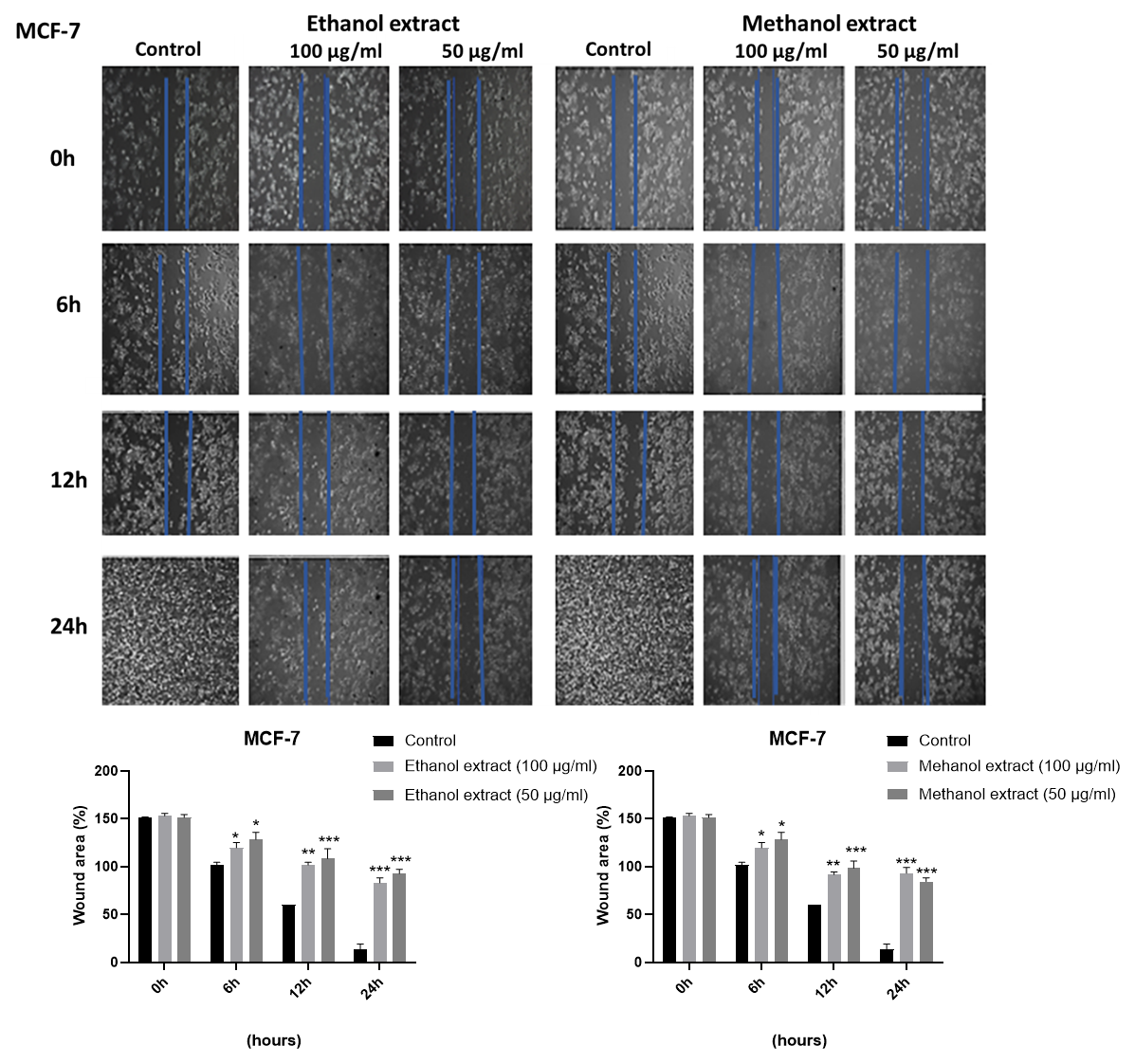


**Fig. S11** Cell migration evaluated by wound-healing assay after treatment with methanol and ethanol extracts of *C. avellana* on MCF-7 cells up to 24 hours. The cell images were taken at 0h, 6h, 12h, 24h. The wound with of views was measured, and the healing width was calculated by wound with at 0 h time point minus wound with at 24 h time point and normalized by control. *Indicates statistically significant differences compared to the control group cells at the same time period *(p<0.05), **(p<0.01), ***(p<0.001). Data are shown as mean ± SD (*n*=3). Microscope objective magnification ×4


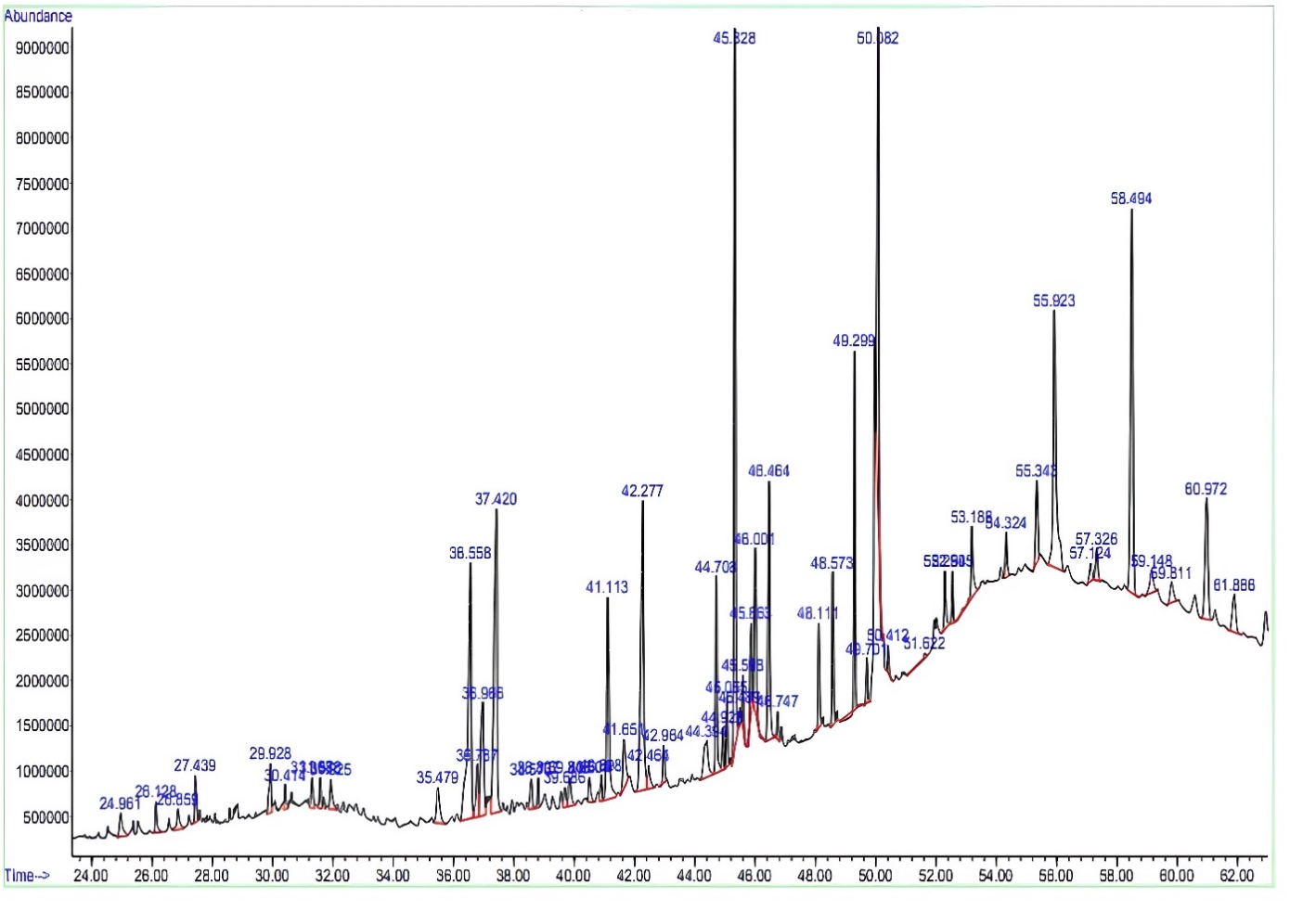
**Fig. S12** Gas chromatography-mass spectrometry (GC-MS) analysis of leaf extract of *C. avellana*

**Table S1** GC–MS profile of methanol extract of *C. avellana* leaf

| No | Peak area% | RT | Name of the compounds | Molecular Formula | Molecular Weight.(g/mol) | | |
| --- | --- | --- | --- | --- | --- | --- | --- |
| 1. | 0.59 | 15.879 | 2,3-Dihydro-3,5-dihydroxy-6-methyl | [C_6_H_8_O_4_](https://pubchem.ncbi.nlm.nih.gov/#query=C6H8O4) | | 144.12 |  |
| 2. | 0.55 | 24.960 | Butanoic acid, ethyl ester | [C_6_H_12_O_2_](https://pubchem.ncbi.nlm.nih.gov/#query=C6H12O2) | | 116.16 |  |
| 3. | 0.42 | 26.130 | 1H-Indene, 1-ethylideneoctahydro-, trans | [C_11_H_18_](https://pubchem.ncbi.nlm.nih.gov/#query=C11H18) | | 150,26 |  |
| 4. | 0.45 | 26.856 | 1,2-Epoxycyclododecane | [C_12_H_22_O](https://pubchem.ncbi.nlm.nih.gov/#query=C12H22O) | | 182.30 |  |
| 5. | 0.56 | 27.441 | Myrtenol | [C_10_H_16_O](https://pubchem.ncbi.nlm.nih.gov/#query=C10H16O) | | 152.23 |  |
| 6. | 0.79 | 29.930 | Dodecanoic acid | [C_12_H_24_O_2_](https://pubchem.ncbi.nlm.nih.gov/#query=C12H24O2) | | 200.32 |  |
| 7. | 0.50 | 31.307 | 3,4,5-Trimethoxyphenol | [C_9_H_12_O_4_](https://pubchem.ncbi.nlm.nih.gov/#query=C9H12O4) | | 184.19 |  |
| 8. | 0.56 | 31.922 | 2-(2-Hydroxyethylamino)pyrimidine | [C_7_H_8_N_4_O](https://pubchem.ncbi.nlm.nih.gov/#query=C7H8N4O) | | 164.16 |  |
| 9. | 0.90 | 35.477 | Trans-coniferyl alcohol | [C_10_H_12_O_3_](https://pubchem.ncbi.nlm.nih.gov/#query=C10H12O3) | | 180.2 |  |
| 10. | 6.59 | 36.558 | 5-Methylhexane-2,4-dione, enol | [C_7_H_12_O_2_](https://pubchem.ncbi.nlm.nih.gov/#query=C7H12O2) | | 128.17 |  |
| 11. | 0.98 | 36.788 | 2-(4-Pentenyl)cyclohexan-1-one | C_11_H_16_O | | 164.24 |  |
| 12. | 2.85 | 36.966 | Grasshopper ketone | C_13_H_22_O | | 224.3 |  |
| 13. | 9.57 | 45.328 | Phytol | [C_20_H_40_O](https://pubchem.ncbi.nlm.nih.gov/#query=C20H40O) | | 296.5 |  |
| 14. | 7.60 | 58.497 | Squalene | [C_30_H_50_](https://pubchem.ncbi.nlm.nih.gov/#query=C30H50) | | 410.7 |  |
| 15. | 7.32 | 37.418 | Orcinol | [C_7_H_8_O_2_](https://pubchem.ncbi.nlm.nih.gov/#query=C7H8O2) | | 124.14 |  |
| 16. | 0.51 | 38.573 | Citronellyl formate | [C_11_H_20_O_2_](https://pubchem.ncbi.nlm.nih.gov/#query=C11H20O2) | | 184.27 |  |
| 17. | 1.03 | 41.654 | Palmitic acid | [C_16_H_32_O_2_](https://pubchem.ncbi.nlm.nih.gov/#query=C16H32O2) | | 256.42 |  |
| 18. | 6.49 | 42.276 | Tetradecanoic acid | [C_14_H_28_O_2_](https://pubchem.ncbi.nlm.nih.gov/#query=C14H28O2) | | 228.37 |  |
| 19. | 0.48 | 42.462 | 1-Butyl-2-ethyloctahydro-4,7-epoxy | [C_8_H_22_O_4_Si_2_](https://pubchem.ncbi.nlm.nih.gov/#query=C8H22O4Si2) | | 238.43 |  |
| 20. | 0.49 | 42.965 | 4-Hydroxy-3,5-dimethoxy-cinnamyl alcohol | [C_11_H_14_O_4_](https://pubchem.ncbi.nlm.nih.gov/#query=C11H14O4) | | 210.23 |  |
| 21. | 1.28 | 44.395 | Oleic acid | [C_18_H_34_O_2_](https://pubchem.ncbi.nlm.nih.gov/#query=C18H34O2) | | 282.5 |  |
| 22. | 2.36 | 44.756 | 1-Octadecene | [C_18_H_36_](https://pubchem.ncbi.nlm.nih.gov/#query=C18H36) | | 252.5 |  |
| 23. | 0.78 | 45.069 | 9,12,15-Octadecatrienoic acid, methyl ester | [C_19_H_32_O_2_](https://pubchem.ncbi.nlm.nih.gov/#query=C19H32O2) | | 292.5 |  |
| 24. | 0.98 | 45.861 | Linoleic acid | [C_18_H_32_O_2_](https://pubchem.ncbi.nlm.nih.gov/#query=C18H32O2) | | 280.4 |  |
| 25. | 2.70 | 46.002 | Linolenic acid | [C_18_H_30_O_2_](https://pubchem.ncbi.nlm.nih.gov/#query=C18H30O2) | | 278.4 |  |
| 26. | 3.84 | 46.461 | Stearic acid | [C_18_H_36_O_2_](https://pubchem.ncbi.nlm.nih.gov/#query=C18H36O2) | | 284.5 |  |
| 27. | 1.21 | 48.113 | N-octadecanoyl-glycine | [C_20_H_39_NO_3_](https://pubchem.ncbi.nlm.nih.gov/#query=C20H39NO3) | | 341.5 |  |
| 28. | 1.71 | 48.572 | 1-Octadecene | [C_18_H_36_](https://pubchem.ncbi.nlm.nih.gov/#query=C18H36) | | 252.5 |  |
| 29. | 3.50 | 49.298 | Arachidic acid methyl ester | [C_21_H_42_O_2_](https://pubchem.ncbi.nlm.nih.gov/#query=C21H42O2) | | 326.6 |  |
| 30. | 6.85 | 50.083 | Arachidic acid | [C_20_H_40_O_2_](https://pubchem.ncbi.nlm.nih.gov/#query=C20H40O2) | | 312.5 |  |
| 31. | 0.54 | 52.542 | Methyl behenate | [C_23_H_46_O_2_](https://pubchem.ncbi.nlm.nih.gov/#query=C23H46O2) | | 354.6 |  |
| 32. | 1.22 | 53.186 | cis-Vaccenic acid | [C_18_H_34_O_2_](https://pubchem.ncbi.nlm.nih.gov/#query=C18H34O2) | | 282.5 |  |
| 33. | 0.64 | 54.327 | 1-Nonedecene | [C_19_H_38_](https://pubchem.ncbi.nlm.nih.gov/#query=C19H38) | | 266.5 |  |
| 34. | 1.38 | 55.342 | 11,13-Dimethyl-12-tetradecen-1-ol acetate | [C_18_H_34_O_2_](https://pubchem.ncbi.nlm.nih.gov/#query=C18H34O2) | | 282.5 |  |
| 35. | 6.26 | 55.927 | Octadecanoic acid, 2,3-dihydroxypropyl ester | [C_25_H_46_O_6_](https://pubchem.ncbi.nlm.nih.gov/#query=C25H46O6) | | 442.6 |  |
| 36. | 0.57 | 57.327 | (Z)-11-Octadecenoic acid | [C_18_H_34_O_2_](https://pubchem.ncbi.nlm.nih.gov/#query=C18H34O2) | | 282.5 |  |
| 37. | 0.60 | 59.149 | Cyclotriacontane | [C_30_H_60_](https://pubchem.ncbi.nlm.nih.gov/#query=C30H60) | | 420.8 |  |
| 38. | 2.82 | 60.971 | Methyl 2-hydroxycyclopentanecarboxylate | [C_7_H_12_O_3_](https://pubchem.ncbi.nlm.nih.gov/#query=C7H12O3) | | 144.17 |  |
| 39. | 1.01 | 61.889 | 1-(2-Furoyl)piperazine | [C_9_H_12_N_2_O_2_](https://pubchem.ncbi.nlm.nih.gov/#query=C9H12N2O2) | | 180.2 |  |
| 40. | 0.64 | 39.847 | OCTADEC-9-ENOIC ACID | [C_18_H_34_O_2_](https://pubchem.ncbi.nlm.nih.gov/#query=C18H34O2) | | 282.5 |  |
| 41. | 3.88 | 41.114 | Methyl palmitate | [C_17_H_34_O_2_](https://pubchem.ncbi.nlm.nih.gov/#query=C17H34O2) | | 270.5 |  |
| 42. | 0.30 | 46.750 | 9-Octadecenoic acid | [C_18_H_34_O_2_](https://pubchem.ncbi.nlm.nih.gov/#query=C18H34O2) | | 282.5 |  |
| 43. | 0.41 | 40.506 | 2,4-Dimethoxybenzyl alcohol | [C_9_H_12_O_3_](https://pubchem.ncbi.nlm.nih.gov/#query=C9H12O3) | | 168.19 |  |
| 44. | 0.36 | 39.684 | 5,7-Dimethyloctahydrocoumarin | [C_11_H_18_O_2_](https://pubchem.ncbi.nlm.nih.gov/#query=C11H18O2) | | 182.26 |  |
|  |  |  |  |  | |  |  |

**Table S2** IC_50_ values of ethanol and methanol extracts of *C. avellana* in cells according to ATP assay results

| \| Cells \| Ethanol extract (μg/ml) \| Methanol extract (μg/ml) \| \| --- \| --- \| --- \| \| MCF-7 \| 32,17 \| 21,08 \| \| MDA-MB-231 \| 32,16 \| 40,16 \| \| A549 \| 20,40 \| 22,04 \| \| H1299 \| 12,04 \| 5,91 \| \| MCF-10A \| >200 \| >200 \| \| BEAS-2B \| >200 \| >200 \| |
| --- | --- | --- | --- | --- | --- | --- | --- | --- | --- | --- | --- | --- | --- | --- | --- | --- | --- | --- | --- | --- | --- |

**Table S3** Selectivity Index (SI) values ethanol and methanol extracts of *C. avellana* in cells according to ATP assay results

| Cells | Ethanol extract | Methanol extract |
| --- | --- | --- |
| MCF-7 | 6,22 | 9,49 |
| MDA-MB-231 | 6,22 | 4,98 |
| A549 | 9,80 | 9,07 |
| H1299 | 16,61 | 33,84 |

**References**

1. Çebi A, Akgün E, Çelikler S, et al (2019) Cytotoxic and genotoxic effects of an endemic plant of Turkey Salvia kronenburgii on breast cancer cell lines. J Cancer Res Ther. https://doi.org/10.4103/jcrt.JCRT_1322_16

2. Tepe Y, Aydın H, Ustaoğlu F, Taştekin Ö (2024) Occurrence and characteristics of PAHs in coastal seawater off the city of Samsun on the Black Sea coast of Turkey. Reg Stud Mar Sci 74:103552. https://doi.org/https://doi.org/10.1016/j.rsma.2024.103552

3. Skehan P, Storeng R, Scudiero D, et al (1990) New colorimetric cytotoxicity assay for anticancer-drug screening. J Natl Cancer Inst. https://doi.org/10.1093/jnci/82.13.1107

4. Andreotti PE, Hartmann DM, Linder D, et al (1995) Chemosensitivity Testing of Human Tumors Using a Microplate Adenosine Triphosphate Luminescence Assay: Clinical Correlation for Cisplatin Resistance of Ovarian Carcinoma. Cancer Res

5. Tabuchi M, Tomioka K, Kawakami T, et al (2010) Serum cytokeratin 18 M30 Antigen Level and Its Correlation with Nutritional Parameters in middle-aged Japanese males with nonalcoholic fatty liver disease (NAFLD). J Nutr Sci Vitaminol (Tokyo). https://doi.org/10.3177/jnsv.56.271

6. Erkisa M, Aydinlik S, Cevatemre B, et al (2020) A promising therapeutic combination for metastatic prostate cancer: Chloroquine as autophagy inhibitor and palladium(II) barbiturate complex. Biochimie 175:159–172. https://doi.org/https://doi.org/10.1016/j.biochi.2020.05.010

7. Genel ME, Adacan K, Selvi S, et al (2024) Apoptosis-inducing, anti-angiogenic and anti-migratory effects of a dinuclear Pd(II) complex on breast cancer: A promising novel compound. Microvasc Res 151:104619. https://doi.org/https://doi.org/10.1016/j.mvr.2023.104619
